# Vitamin A resolves lineage plasticity to orchestrate stem cell lineage choices

**DOI:** 10.1126/science.adi7342

**Published:** 2024-03-08

**Authors:** Matthew T Tierney, Lisa Polak, Yihao Yang, Merve Deniz Abdusselamoglu, Inwha Baek, Katherine S Stewart, Elaine Fuchs

**Affiliations:** Howard Hughes Medical Institute, Robin Chemers Neustein Laboratory of Mammalian Cell Biology and Development, The Rockefeller University; New York, NY 10065, USA.

## Abstract

Lineage plasticity – a state of dual fate expression – is required to release stem cells from their niche constraints and redirect them to tissue compartments where they are most needed. In this work, we found that without resolving lineage plasticity, skin stem cells cannot effectively generate each lineage in vitro nor regrow hair and repair wounded epidermis in vivo. A small molecule screen unearthed retinoic acid as a critical regulator. Combining high-throughput approaches, cell culture and in vivo mouse genetics, we dissected its roles in tissue regeneration. We found that retinoic acid is made locally in hair follicle stem cell niches, where its levels determine identity and usage. Our findings have therapeutic implications for hair growth as well as chronic wounds and cancers where lineage plasticity is unresolved.

## One-Sentence Summary

By regulating lineage plasticity, retinoids dictate whether and when bipotent adult stem cells pursue one cell fate versus another.

Adult stem cells reside in anatomically distinct niches that reliably guide self-renewal and the production of each differentiated cell type (1, 2). However, injury frequently disrupts this homeostasis as stem cells are released from their normal constraints and repurposed to regenerate tissue compartments that they have not made since embryogenesis. To transition from one developmental lineage to another and repair the damaged area, stem cells must first enter a transient but obligatory state of lineage plasticity (3, 4).

Lineage plasticity is defined by the dual expression of cell identity transcription factors of both new and prior lineages and is typical of wound-induced stress pathways that bestow stem cells with fate flexibility (4). This state has been observed across multiple tissues in regeneration-inducing settings, including wounding, neighboring cell death, and transplantation (5–9). In healthy tissues, stem cells ultimately either return to their prior niche-defined trajectory or undergo a fate change upon entering a different microenvironment. How lineage plasticity is resolved is important to understand, because conditions that perpetuate it can lead to chronic wound states or tumorigenesis (10).

To tackle this key issue, we turned to the mammalian skin epithelium – a barrier tissue that has evolved to rapidly heal in response to environmental insults and that contains several discrete stem cell populations derived from a common embryonic progenitor (11). During homeostasis, interactions with their niches specialize the tasks that each stem cell performs. Hair follicle stem cells (HFSCs) within the so-called ‘bulge’ niche constitute the engine for cyclical bouts of hair regeneration, whereas epidermal stem cells (EpSCs) within the basal layer of the interfollicular epidermis generate the flux of upward stratifying cells that rejuvenate the skin’s barrier to the outside world.

Depending on the depth of injury, different proportions of HFSCs and EpSCs will migrate out of their niches and enter a state of lineage plasticity to re-epithelialize the denuded epidermis (12–14). Lineage plasticity in skin is typified by the co-expression of *Sox9* and *Klf5,* encoding master regulatory transcription factors that distinguish HFSCs and EpSCs, respectively (4). Notably, HFSCs undergo a fate switch while repairing the epidermis in every-day abrasion wounds and upon autologous split-thickness skin grafting, which is the gold standard of care for severely burned patients (14, 15).

HFSCs enter a similar wound-like state when placed in serum and growth factor-rich culture media, which prompts the co-expression of SOX9 and KLF5 and initiates widespread chromatin remodeling characteristic of lineage plasticity (16). We exploited this phenomenon and used the mouse as a model system to screen for factors able to resolve this plasticity and unravel how its functions in homeostasis, lineage specification, and wound repair.

## A functional screen to resolve lineage plasticity in cultured HFSCs

A master regulator of EpSCs, KLF5 is transiently induced by HFSCs in response to injury as they exit their niche, migrate upward into the wound site, and participate in epidermal repair (14). KLF5 becomes a hallmark of lineage plasticity when activated in wound-stimulated HFSCs, which dually maintain low levels of SOX9 (4). To restore homeostasis and resume hair regeneration, HFSCs suppress KLF5 and the genes that it regulates while elevating SOX9 to homeostatic levels. We sought to determine whether resolving lineage plasticity is essential to preserve stem cell identity, and if so, what are the factors that control it.

**Fig. 1:**
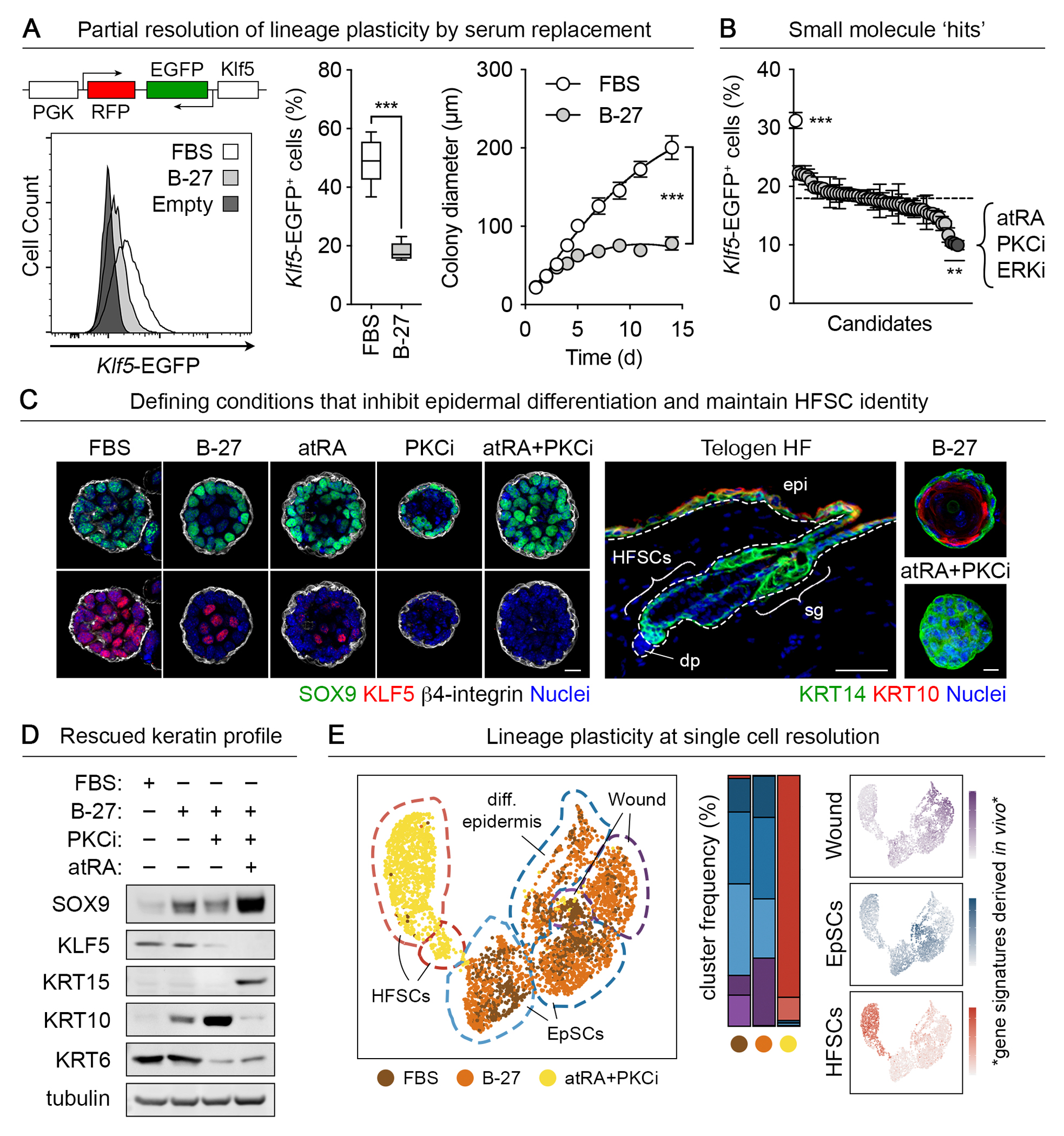
A screen to resolve lineage plasticity in tissue stem cells ex vivo. (**A**) Cultured HFSCs were stably transduced with a lentiviral Klf5-EGFP reporter (left). Flow cytometry quantification (center) showed that serum replacement reduced Klf5 reporter expression in 3D cultures. Colony diameter plateaued over time in serum replacement media, in contrast to unrestrained outgrowth in serum-replete media (right) (n=5 technical replicates across three purified HFSC lines). (**B**) Quantification of Klf5-EGFP^+^ cells after screening a library of small molecules implicated in wound repair identified several small molecule ‘hits’ (n=5). The dashed line indicates baseline EGFP levels in B-27 media with vehicle-only controls. The significant hits that repressed Klf5-EGFP were: atRA, the PKCa/b inhibitor Gö6976, and FR180204, a competitive inhibitor of ERK1/2. (**C and D**) Immunofluorescence (C) and immunoblot (D) analyses show that serum replacement and treatment with atRA and PKCi (n=6) resolves lineage plasticity, as judged by elevated HFSC master regulator SOX9 and silenced EpSC transcriptional regulator KLF5. Keratin profiles reflect the loss of epidermal differentiation and wound-induced suprabasal markers (KRT10, KRT6), uniform expression of pan-skin progenitor marker KRT14, and gain of KRT15, a HFSC marker. The epidermal-dermal interface of sagittal skin section is delineated by a dashed line. epi, epidermis; sg, sweat gland; dp, dermal papilla. Scale bar, 20μm. (**E**) UMAP representation and unsupervised k-nearest-neighbor-based clustering of single cell transcriptomes from 3,757 cultured HFSCs reveal that RA-PKCi co-treatment groups cells in near-uniform clusters distinct from wound-like and epidermal cell types (left, center). Annotated cluster assignments made using gene signatures derived from independent bulk sequencing of in vivo HFSCs, EpSCs, and lineage traced HFSCs isolated at intermediate stages of wound repair confirmed that atRA+PKCi-treated cells had a strong HFSC signature and reduced EpSC and wound signatures (right).

To monitor lineage plasticity, we generated a reporter system. Purified HFSCs (12) were stably transduced by lentivirus harboring a *Klf5-EGFP-NLS* transgene (EGFP, enhanced green fluorescent protein) with a constitutive *Pgk*-driven red fluorescent protein (RFP)-NLS transduction control (Fig. 1A). When grown in a modified three dimensional (3D) culture system in which fetal bovine serum (FBS) was replaced with a serum-free media supplement (B-27) (17), *Klf5*-EGFP levels were partially reduced and colony growth was stabilized without compromising colony forming efficiency (fig. S1, A and B). Notably, serum replacement was not sufficient to reduce *Klf5*-EGFP levels without antioxidants, whereas media formulated to mimic human plasma accomplished this even in the presence of serum (fig. S1C). These results underscored the complexity of signaling networks involved in suppressing lineage plasticity.

To further reduce *Klf5*-EGFP levels in serum-free conditions, we targeted pathways associated with lineage plasticity. Initially focusing on the AP-1, ETS, and STAT families (4), we examined selective inhibitors to these pathways, but did not observe reduction of *Klf5*-EGFP levels without negatively affecting colony formation (fig. S1, D and E). Similarly, we tested agonists and antagonists of core signaling cascades involved in lineage progression during hair regeneration, including members of the WNT (Wingless-related integration site), TGFβ (transforming growth factor-beta), BMP (bone morphogenetic protein) and SHH (sonic hedgehog) pathways (1). All failed to significantly affect *Klf5*-EGFP levels or resolve lineage plasticity (fig. S1E).

To home in on the elusive mediators of lineage plasticity, we screened HFSCs cultured in serum-free conditions with a library of small molecules that have been broadly implicated in wounding. Of the >70 compounds tested (Table S1), three appreciably diminished *Klf5*-EGFP levels: (i) ERKi, a competitive inhibitor of extracellular signal-regulated kinases (ERKs) and mitogen activated protein kinases (MAPKs); (ii) PKCi, an inhibitor of calcium-dependent protein kinase C (PKC) and (iii) all-trans-retinoic acid (atRA), the major active metabolite of retinol and vitamin A_1_ in skin (Fig. 1B). Despite prior implications in wound repair (PKC and ERK) (18–21) and hair growth (ERK and atRA) (22, 23), their precise roles in these processes have remained ambiguous. Given the role of the ERK-MAPK pathway in normal hair cycling (24), we turned our attention instead to PKCi and atRA.

We explored these two hits against a backdrop of serum-free conditions, where SOX9 and KLF5 had already partitioned their expression peripherally and internally within colonies, respectively (Fig. 1C). Although atRA was not as effective as PKCi in reducing KLF5 expression, it markedly expanded the SOX9 population. The combination of atRA and PKCi optimally resolved lineage plasticity and favored a HFSC fate, which was particularly evident in 3D culture conditions that mimicked the *in vivo* microenvironment (fig. S2, A and B). Keratin-6 (KRT6) and *Ets2*, hallmarks of hyperproliferative and cancerous epidermis (25, 26), were suppressed by atRA and PKCi (Fig. 1D and fig. S2C). Prominent HFSC identity markers SOX9 and KRT15 were elevated markedly by atRA, which correspondingly suppressed epidermal markers including SCA1 and KRT10 (Fig. 1D and fig. S2, D and E) (1). The uniform expression of KRT14, a universal marker of epithelial skin progenitors, further reflected the stemness of these cultures as did the expression of *Nfib* and *Tbx1*, whose encoded transcription factors are known to help to maintain the HFSC epigenetic landscape and support self-renewal (27–29) (fig. S2F).

The ability to resolve lineage plasticity and achieve a HFSC identity in culture was exemplified more globally through single cell RNA-sequencing (scRNA-seq) (Fig. 1E). Leveraging both existing bulk RNA-seq data from EpSCs and lineage-traced HFSCs isolated before and during wound repair in vivo, we obtained individual gene signatures for each stem cell type and condition (4) and measured each in our optimized cultures (fig. S3A). HFSCs propagated with atRA and PKCi formed a homogeneous cluster with a strong in vivo HFSC signature and nearly absent of wound or EpSC signatures (Fig. 1E). Notably, signature genes associated with active enhancers in skin squamous cell carcinomas (SCCs) (26) were also significantly reduced in the HFSC cluster (fig. S3B), underscoring the overlapping roles of atRA-mediated lineage plasticity antagonism in wounds and cancer (4).

Taken together, our screen unearthed a powerful set of factors governing the behavior of these multipotent tissue stem cells, specifically towards resolving lineage plasticity and maintaining a HFSC identity.

## atRA acts at the level of nuclear retinoid receptors to directly resolve lineage plasticity.

Our results suggested that atRA may act specifically at the chromatin level to regulate HFSC fate during wound repair. To test this possibility, we first sought to understand how atRA availability might be controlled locally in the skin. We found expression patterns and activity levels of the rate-limiting enzymes for atRA synthesis (30) in the HFSC niche and in purified HFSCs [aldehyde dehydrogenase 1 (ALDH1) isoforms, fig. S4A]. To directly test the consequences of this activity, we transduced HFSCs with a lentivirus harboring constitutively active H2B-GFP and an RFP reporter driven by a retinoic acid response element (*RARE*), bound by retinoic acid receptor (RAR) retinoid X receptor (RXR) heterodimers and activated when retinoic acid is present (31, 32). *RARE*-RFP activity was stimulated in cultured HFSCs by both atRA and its upstream substrate retinol (ROL), or vitamin A – the circulating form of retinoids delivered to tissues – and was abrogated by an ALDH1A inhibitor (fig. S4B). Thus, HFSCs are equipped with the cellular machinery to take up retinol and convert it to active atRA, and HFSCs have the requisite atRA-responsive nuclear retinoid receptors to drive transcription.

**Fig. 2:**
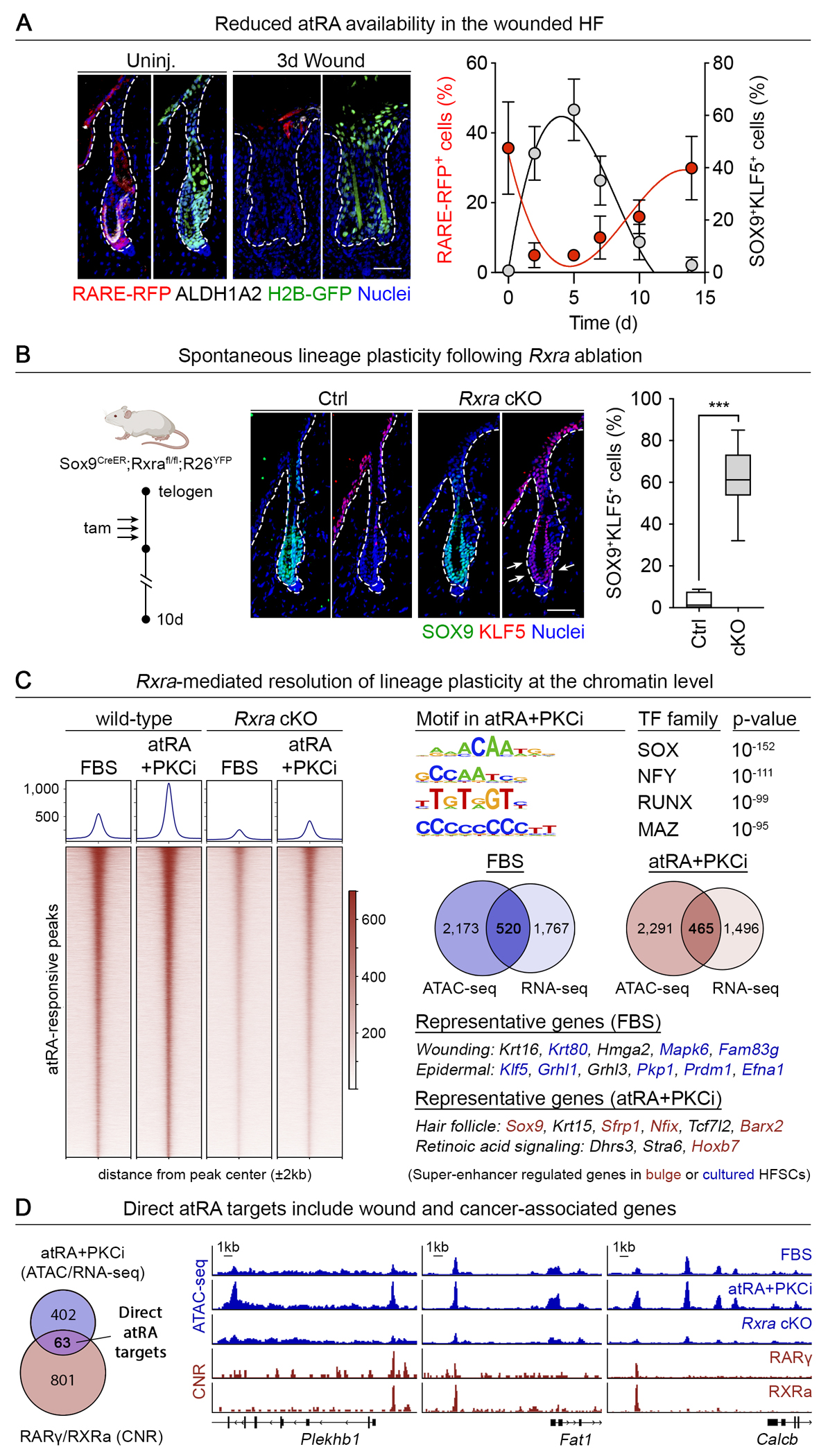
Retinoic acid acts at the chromatin level to resolve lineage plasticity. (**A**) atRA-RAR/RXR activity is detected in the HFSC niche *in vivo* as judged by a retinoic acid response element reporter (*RARE*-RFP; H2B-GFP transduction control) in adult skin epithelium. ALDH1A2 immunolabeling confirms the co-presence of this rate-limiting atRA production enzyme. Both are transiently lost after an abrasion wound (left). The epidermal-dermal interface is delineated by a dashed line. Scale bar, 50μm. The kinetics of *RARE*-RFP activity during wound repair was measured by flow cytometry (n=5). Lineage plasticity (percentage of SOX9^+^KLF5^+^ cells in the HF) is overlaid (right; n=10–12 HFs per mouse, n=8 mice). Uninj., uninjured. (**B**) Conditional *Rxra* ablation in *Sox9*-CreER^+^ HFSCs during telogen results in ectopic KLF5 expression. The epidermal-dermal interface is delineated by dashed lines. Arrows denote double immunolabeled SOX9^+^KLF5^+^ cells, reflective of lineage plasticity and quantified at right (n=5). Scale bar, 50μm. Ctrl., control. (**C**) Line plots and heatmap of ATAC-seq signal intensity in atRA-stimulated chromatin peaks from wild-type and *Rxra* cKO HFSCs cultured in FBS or atRA+PKCi media (left; n=2). HOMER motif analysis of *Rxra*-dependent chromatin domains differentially opened in atRA+PKCi media (top right). Note that atRA-sensitive peaks are highly enriched for SOX motifs. Venn diagrams of overlapping (opened or closed) *Rxra*-dependent chromatin peaks (ATAC-seq) and DEGs (RNA-seq) (bottom right). Selected genes are listed by culture condition, color-coded if previously determined to be a super-enhancer regulated gene in cultured or *in vivo* HFSCs (16). Notable among these *Rxra*-dependent, atRA-regulated genes is *Sox9,* encoding a pioneer factor essential for activating key HFSC genes directly and silencing EpSC genes indirectly (34). (**D**) Venn diagram showing transcripts and accessible chromatin peaks that are atRA and RXRa sensitive and co-bound by RARγ and RXRa (left). Representative ATAC peaks and CNR binding profiles for RARγ and RXRa at direct atRA target genes (right).

Next, we used our in utero lentiviral delivery system (33) to stably transduce the adult skin epithelium with our *RARE*-RFP reporter and detected activity in HFSCs and their niche adjacent to ALDH1A^+^ cells *in vivo* (Fig. 2A). Both reporter activity and ALDH1A levels dropped upon injury, concurrent with the induction of lineage plasticity (Fig. 2A and fig. S4C). The kinetics of the wound response monitored by immunofluorescence microscopy indicated that by 2 weeks after injury, *RARE*-RFP activity had resumed within the HFSC niche and the number of SOX9^+^KLF5^+^ cells had waned (Fig. 2A). These results exposed a marked inverse correlation between atRA availability and lineage plasticity during wound repair.

Given the broad effects of retinoids on skin biology, we next addressed whether atRA-mediated activation of the RAR autonomously regulates lineage plasticity in HFSCs *in vivo.* To this end, we generated *Sox9CreER;Rxra-fl/fl;Rosa26-LSL-YFP* mice to conditionally delete the retinoid X receptor *Rxra*, an obligate heterodimeric partner of RAR. *Rxra* ablation in HFSCs during the resting phase of the hair cycle led to the spontaneous induction of lineage plasticity even in the absence of a wound (Fig. 2B), providing evidence that atRA signaling acts at the chromatin level to suppress lineage plasticity and maintain HFSC identity.

To mechanistically discern how atRA and its RXR/RAR transcriptional effector accomplishes this feat, we purified HFSCs from wild-type and conditionally targeted [*Rxra* conditional knockout (cKO)] mice and cultured them in conditions that either perpetuate lineage plasticity (FBS) or resolve this state (atRA+PKCi). As judged by ATAC-seq (assay for transposase-accessible chromatin using sequencing), 54 and 43% of chromatin regions that had gained or lost accessibility during lineage plasticity resolution, respectively, were sensitive to *Rxra* ablation (Fig. 2C and fig. S5, A to C). Motifs for the SOX family were particularly enriched in newly opened, *Rxra*-dependent domains (Fig. 2C). Moreover, *Sox9* was upregulated by atRA in a dose-dependent manner, which suggests that SOX9 acts downstream of atRA-dependent nuclear receptor signaling (fig. S5D).

The atRA sensitivity of SOX9 was intriguing given that SOX9 acts as a pioneer factor at key HFSC ‘super-enhancers’ known to regulate HFSC identity genes (16). In this regard, a cohort of in vivo HFSC super-enhancer regulated genes(16) were upregulated by atRA in a *Rxra*-dependent manner (Fig. 2C and fig. S5, E and F). This included *Nfix*, whose loss in HFSCs also results in spontaneous lineage plasticity (28). Conversely, wound-related and epidermal differentiation genes, including *Klf5* and *Grhl1/3,* were strongly downregulated, consistent with SOX9’s established role in silencing EpSC enhancers (34).

Turning to CNR (cleavage under targets and release using nuclease), we assessed the chromatin landscape of cultured HFSCs for the binding of RXRα and RARγ, the most likely retinoid heterodimer for HFSCs in vivo (fig. S5G) (35). A set of direct atRA target genes were identified (Fig. 2D), including known atRA targets (e.g. *Plekhb1*), positive regulators of a HFSC fate (e.g. *Sox9 and Tcf7l2*), and negative regulators of wound- and cancer-related stem cell behavior (e.g. *Fat1* and *Calcb*) (36–38). Thus, atRA acts at the chromatin level to resolve lineage plasticity by promoting HFSC identity while concomitantly silencing the EpSC fate.

## atRA functions with BMPs and WNTs to regulate HFSC identity and lineage progression

Our chromatin landscaping revealed that although atRA signaling was essential to resolve lineage plasticity and shift HFSCs to a state more characteristic of in vivo homeostasis, atRA was not sufficient on its own to fully recapitulate in vivo HFSC features. Given our newfound ability to resolve lineage plasticity *ex vivo*, we turned to addressing how atRA interacts with other signaling pathways that are known to govern physiological HFSC behavior.

**Fig. 3:**
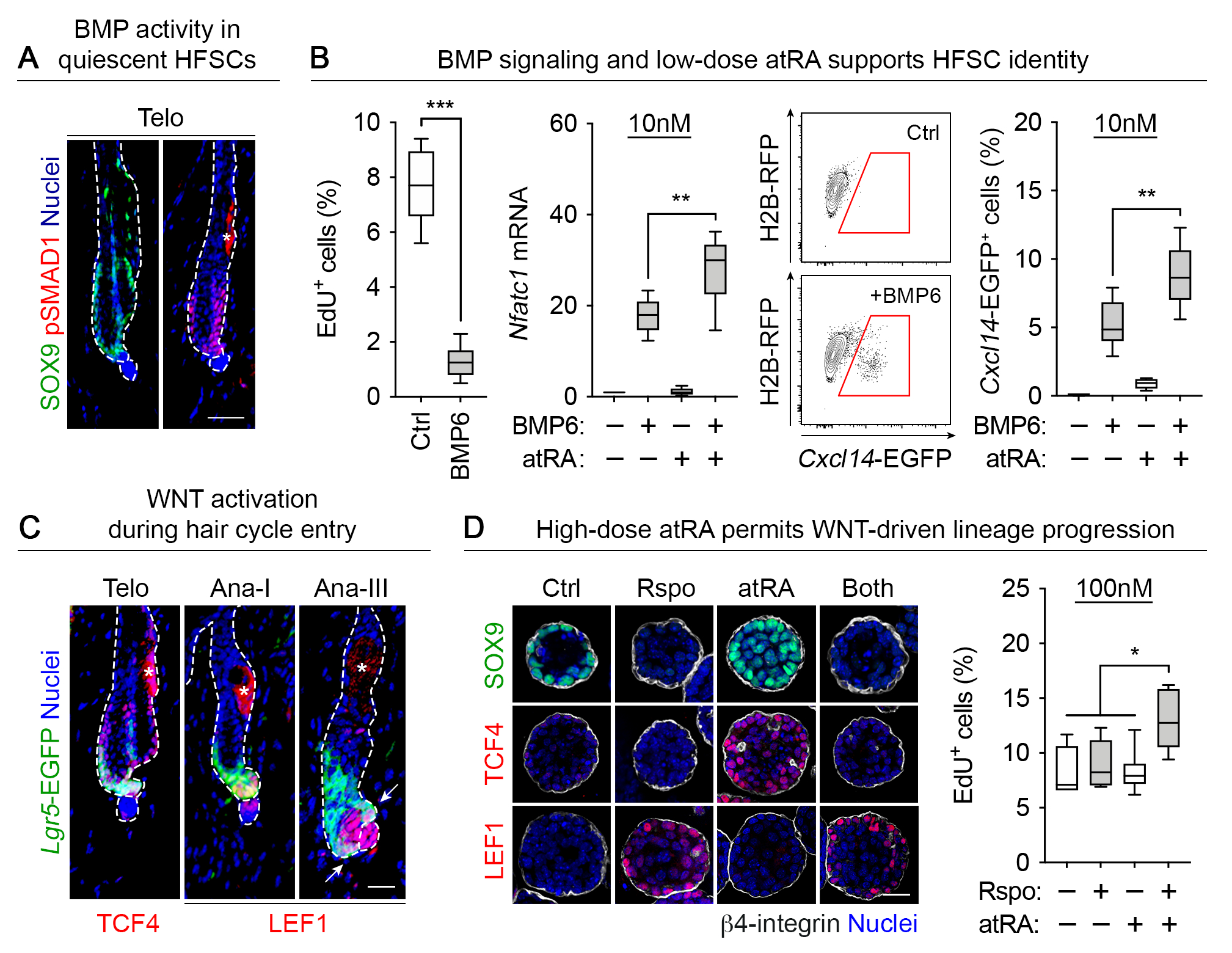
atRA cooperates in a dose-dependent manner with BMPs and WNTs to influence the balance between HFSC quiescence and activation. (**A**) BMP activity in SOX9^+^ quiescent bulge HFSCs *in vivo*. The epidermal-dermal interface is denoted by dashed lines; asterisk refers to autofluorescence in the sebaceous gland. Scale bar, 50μm. (**B**) The percentage of EdU^+^ cultured HFSCs is reduced by BMP6 (left; n=6). Quiescent HFSC markers *Nfatc1* (center) and HFSC-associated *Cxcl14-EGFP* super-enhancer reporter activity (right) are induced by co-treating with BMP6 and low-dose RA (10 nM). Gates outlined in red denote EGFP^+^ cells (n=5–6). (**C**) During telogen, TCF4 and *Lgr5*-EGFP are expressed in HFSCs. Upon hair cycle induction, *Lgr5*-EGFP tracks with the ORS while LEF1 is upregulated in TACS and lower ORS (arrows). The epidermal-dermal interface is denoted by dashed lines; asterisks refer to autofluorescence in the sebaceous gland. Scale bar, 50μm. (**D**) The TCF4:LEF1 switch is accurately modeled in atRA-treated cultured HFSCs by exposure to Rspo, with associated SOX9 downregulation (left). Scale bar, 50μm. High-dose atRA (100 nM) is required for a Rspo-dependent increase in cultured HFSC proliferation, quantified by flow cytometry (right; n=6).

To this end, BMPs are produced by the inner ‘bulge’ layer of the HFSC niche and are responsible for maintaining quiescence (39–41) (Fig. 3A). Notably, although atRA was permissive for HFSC proliferation, the addition of BMPs overrode this response and triggered cell cycle exit (Fig. 3B and fig. S6A). BMP6 and atRA together showed the most potent effect as judged by quiescence markers *Nfatc1* (42) and a *Cxcl14-eGFP* reporter, both of which are also active in quiescent HFSCs in vivo but not in serum/growth factor-rich conditions in vitro (19) (fig. S6B). BMP6’s effects were optimal at low (10nM) atRA levels (Fig. 3B and fig. S6C). By contrast, PKCi exerted little or no effect on these parameters. These data imply that by suppressing lineage plasticity and poising chromatin for HFSC identity, atRA establishes the necessary foundation for working cooperatively with another niche factor, BMP, to accentuate quiescence and maintain stemness.

By contrast, the transition from HFSC quiescence to activation at the start of a new hair cycle is known to be controlled by BMP inhibitors and activators of canonical WNT signaling (43), induced through crosstalk with the nearby specialized mesenchyme called the dermal papilla (DP) (43). Once activated, HFSCs produce two progenitor populations: SOX9^+^TCF3/4^+^ cells in the upper outer root sheath (ORS), which harbors the HFSCs to be used for the next hair cycle (41), and short-lived, highly proliferative LEF1^+^TCF1^+^ transit-amplifying cells’(TACs) that maintain contact with the DP and ultimately give rise to the hair shaft (HS) and its channel, the inner root sheath (IRS) (44–46) (Fig. 3C).

Mimicking a wound state, none of the four WNT-regulating TCF or LEF transcription factors were expressed under standard serum conditions in vitro (16) or in serum-free conditions, even with PKCi. The coordinated effects of atRA and BMP6 on HFSC maintenance were consistent with their ability to increase TCF3/4 levels, raising the threshold for WNT activation (Fig. 3D and fig. S7A) (45). Conversely, R-spondins (Rspo) (47) mediated a canonical WNT-signaling switch between stemness and TACs by promoting proliferation only in the presence of atRA, simultaneously downregulating SOX9 and TCF3/4 and inducing LEF1 and TCF1 (45) (Fig. 3D and fig. S7, B to D). In this setting, the switch from quiescence to proliferation was optimal when Rspo was combined with a 10-fold higher atRA concentration (100nM) than was optimal for maintaining quiescence (Fig. 3D and fig. S7E).

These data suggested that although atRA is essential for maintaining a HFSC identity, its relative levels determine whether it will optimally function to maintain quiescence (low levels) or mediate the early stages of hair follicle (HF) regeneration (high levels). To test this *in vivo*, we topically applied atRA to the back skin of mice during the resting phase of the hair cycle and monitored hair cycle entry (48). atRA facilitated hair cycle entry in a dose-dependent manner, increasing 5-ethynyl-2’-deoxyuridine (EdU) incorporation in CD34^+^ HFSCs and accelerating HF downgrowth at early time points after administration (fig. S8A). This was abrogated by topical treatment with an inhibitor of RAR signaling (RARi). Accordingly, we observed an atRA-dependent delay in hair cycling when placing mice on a vitamin A-deficient diet, confirmed to deplete the skin of retinol without affecting body mass (fig. S8, B and C). Taken together, these data were consistent with and add mechanistic insights into prior observations that differences in retinoid levels affect hair growth (49–52).

## Directed differentiation to HF lineages via combinatorial niche signaling

In vivo, a myriad of secondary circuits and intercellular interactions operate within the native microenvironment of the skin and participate in hair regeneration. By suppressing the epidermal fate and resolving lineage plasticity, retinoids provided a culture platform for teasing apart the complex lineage signals that directly affect HFSCs and specify the spatiotemporally defined concentric lineages of the fully grown HF.

**Fig. 4:**
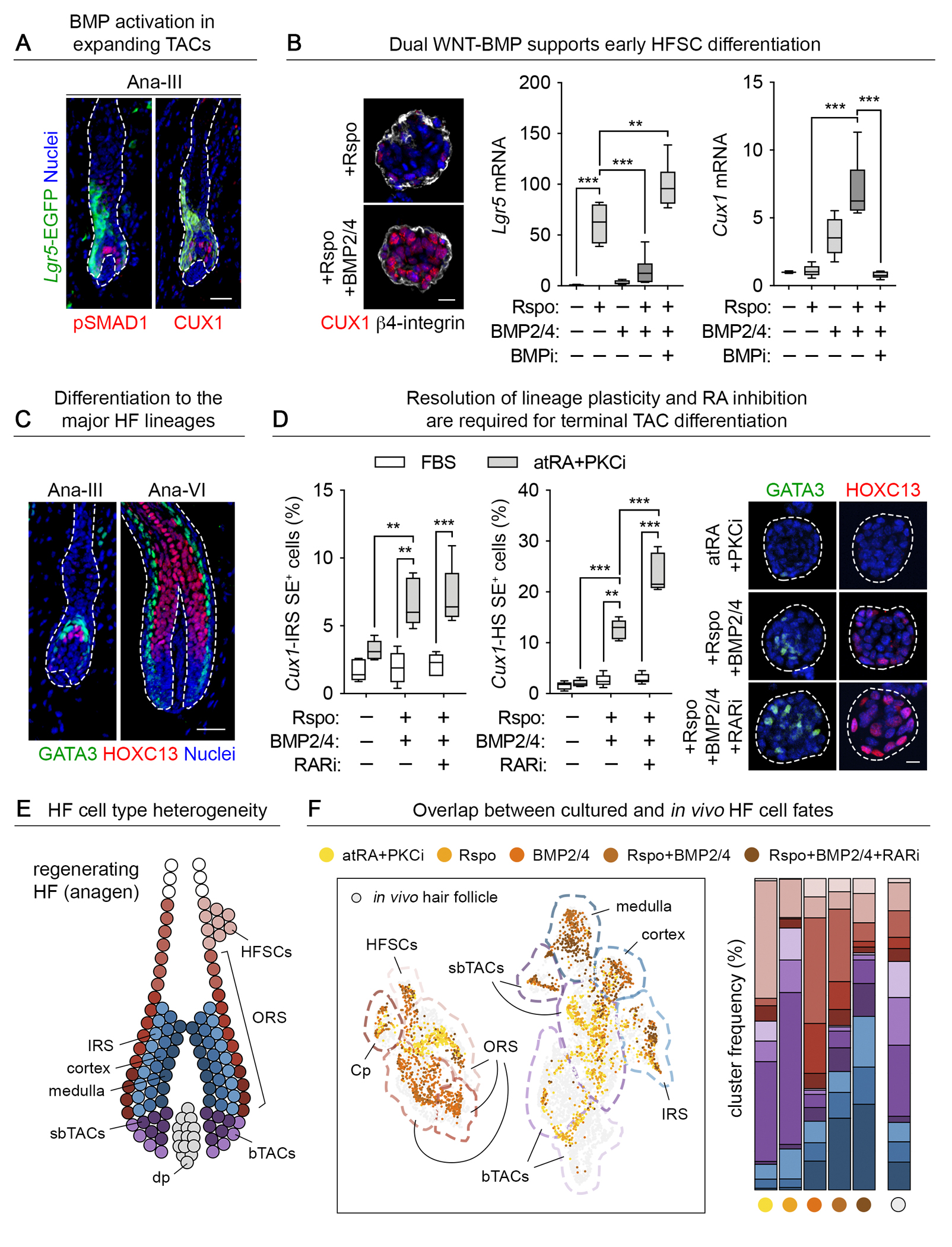
atRA-mediated directed differentiation to HF lineages via combinatorial niche signaling. (**A**) Downstream of WNT-activated HFSCs in the early anagen phase of the hair cycle, BMP signaling (measured by pSMAD1 immunolabeling) distinguishes CUX1^+^ TACs of the hair ‘bulb’ and is mutually exclusive to *Lgr5*-EGFP in the ORS. The epidermal-dermal interface is denoted by dashed lines. Scale bar, 50μm. (**B**) atRA-treated cultured HFSCs respond similarly: BMPs upregulate *Cux1* in Rspo-stimulated HFSCs while antagonizing *Lgr5*-EGFP activity (n=6). Scale bar, 20μm. (**C**) In full or late anagen, the HOXC13^+^ HS and GATA3^+^ channel IRS lineages emerge. The epidermal-dermal interface is denoted by dashed lines. Scale bar, 50μm. (**D**) In culture, only when lineage plasticity (FBS) is resolved (atRA+PKCi) are HFSCs able to cooperate with WNT and BMP signals and activate these downstream hair lineages. Note that the HS lineage is further promoted by retinoic acid receptor inhibition (RARi). Quantification is by flow cytometry for reporter activity driven by *Cux1* enhancer elements active in the IRS and HS, respectively (16) (n=5). GATA3 and HOXC13 immunofluorescence are shown at right. HFSC colonies are outlined by white dashed lines. Scale bar, 20μm. (**E**) Scheme depicting the HFSC-derived cell fates of the anagen-phase hair follicle. (**F**) UMAP clustering of cultured HFSCs treated with atRA and PKCi and additionally exposed to Rspo, BMP or RARi as noted. Harmony (57) was used to place these data in the context of a reference dataset of autologous, FACS-purified cells from skin HFs (left; 5,333 cultured cells, 6,007 *in vivo* HF cells). Shifts in cultured HFSC identity, mapped onto annotated clusters, were consistent with the establishment of traits of HFSC or ORS, Rspo-activated TACs and differentiating IRS or HS lineages after WNT-BMP co-stimulation, respectively (right).

As the hair cycle progresses in vivo, WNT-active proliferating cells bifurcate into the expanding *Lgr5*^+^ ORS and more committed *Cux1*^*+*^*Lgr5*^*neg*^*Lef1*^*+*^ TACs. Although *Lgr5* and *Lef1/Tcf1* are canonical WNT targets (53, 54), their expression in vitro and in the HF were mutually exclusive (Fig. 3C and Fig. 4A). Using our culture system, we dissected the underlying reasons. Both *Lef1* and *Cux1* were sensitive to WNT signaling (fig. S9, A and B). However, *Cux1* upregulation was optimal under BMP^hi^ conditions, thereby bifurcating progenitors into the discrete lineages of the growing HF (44, 55) (Fig. 4B). By contrast, *Lgr5-EGFP* required BMP inhibition and atRA for optimal activity alongside WNT stimulation (Fig. 4B and fig. S9C). These findings suggested that through reliance upon multiple signaling inputs, the expression of WNT target genes can be tailored to suit the particular needs of different stem cell niches.

Using our culture system to disentangle the signals critical for distinguishing HS and IRS differentiation, we took advantage of distinct *Cux1*-associated enhancer elements that faithfully drive reporter activity in these separate fates downstream of TACs and upstream of hair formation (45) (Fig. 4C and fig. S9D). Using these reporters, we consistently found that the resolution of lineage plasticity in cultured HFSCs was required to specify TACs and downstream progenitors responsible for making the HS and IRS lineages that are marked by their transcription factors HOXC13 and GATA3, respectively (Fig. 4D). BMP2 and BMP4 were most effective in upregulating reporter activity (fig. S9E). By contrast, conditions that perpetuate lineage plasticity were incompatible with differentiation to any hair lineage (Fig. 4D). Within colonies where dual IRS and HS differentiation was observed, spatial organization emerged as it does *in vivo*, with GATA3 (IRS) localized more centrally while HOXC13 (HS) was enriched in peripheral cells (fig. S9F).

Notably, further lineage restriction to specify the HS and its channel was achieved by inhibiting RAR-RXR receptor activity (Fig. 4D). Taking our findings to an in vivo setting, we applied atRA or RARi to the back skins of mice at mid-anagen, when the HS and IRS begin to form (Fig. 4C). Topical atRA substantially reduced the size of the hair bulb overall, reduced the relative proportion of HOXC13^+^ to GATA3^+^ cells, and impeded hair cycling (fig. S10A). Thus, although atRA is required to promote HFSC specification and enable lineage progression, proper formation of the HS relies upon downstream atRA repression.

By reconstituting lineage-specified cell populations in vitro, we could assess their in vivo regenerative potential. Yellow fluorescent protein (YFP)-labeled progenitors from each group were combined with allogenic dermal fibroblasts and transplanted onto mouse back skins (56). Upon engraftment, our HFSCs efficiently produced hair when they had been cultured in serum-free media primed with either atRA+PKCi alone, atRA+PKCi+BMP6 or atRA+PKCi+Rspo (Fig. S10B). However, if HFSCs were co-treated with Rspo and BMP2/4, with or without RAR inhibitors, hair production was abolished. This result indicated that cell populations that had advanced beyond the progenitor state were irreversibly committed to differentiation and were defective at hair regeneration.

Altogether, these findings underscored the value of our system in dissecting complex signaling intersections that occur within HFSCs as they transition from quiescence to activation to differentiation. In particular, we elucidated the importance of resolving HFSC lineage plasticity as a prerequisite to generating a microenvironment conducive to receiving the multifaceted signals required to maintain HFSCs and to orchestrate their progression along discrete lineage pathways.

## Mapping cultured HFSCs to the in vivo HF with single cell transcriptomics

To more rigorously evaluate the similarities between the HF lineage program *in vivo* and that produced by our stepwise differentiation program *in vitro*, we again turned to scRNA-seq. For a reference dataset, we used transcriptomes of cells purified by fluorescence activated cell sorting (FACS) of autologous anagen-phase HFs. We then evaluated the characteristics of every culture-derived HF cell type after their integration with Harmony, an algorithm for performing integration of single cell genomics datasets (57).

Clustering after dimensional reduction by ‘uniform manifold approximation and projection’ (UMAP) identified 12 annotated cell types that captured the full complexity of HF fates (Fig. 4, E and F and fig. S11A). Similar results were achieved using cluster assignments and prediction scores calculated with a weighted nearest neighbor approach (58) (fig. S11B). Cultured HFSCs exposed to atRA and PKCi predominantly clustered with the upper ORS (uORS), analogous to the ‘bulge’ and known to retain stemness across hair cycles (41). HFSCs exposed to Rspo were more similar to shorter-lived TACs, whereas HFSCs treated with Rspo and BMPs corresponded to the major lineages of mature HFs (46).

As judged by Monocle3 (59), an analysis toolkit to evaluate RNA-seq data and infer the trajectory of differentiation, our cultured HFSCs followed an orderly route by advancing through progenitor states and terminating in the robust induction of HS genes (fig. S11C). BMPs drove positioning along this axis, advancing cells to later stages of pseudotime. Differentially expressed gene (DEG) analysis identified ‘signature genes’ and biological processes previously associated with each HF cell type (60), corroborating the fidelity of our protocols (fig. S11D). Overall, our ability to define conditions that achieve lineage specification and differentiation of HFSCs outside of their native niches suggests that soluble signals are central to lineage specification.

Seeking potential mechanisms underlying stem cell fate decisions, we performed pathway enrichment analysis on our cultured HFSCs relative to HFSCs in their native niche. Epidermal differentiation was among the most notable features prevented by atRA and PKCi and in line with in vivo HFSCs (fig. S12). By contrast, extracellular matrix (ECM) assembly, global translation, and glycolysis were all enriched in cultured stem cells, whereas HFSCs in vivo maintain ECM at steady state levels (61, 62), low rates of translation (63, 64), and rewire metabolic networks (65). These differences likely reflected the need for HFSCs in vitro to overcome these constraints to self-renew and expand.

## atRA functions in wound repair by regulating the dynamics of lineage plasticity

**Fig. 5:**
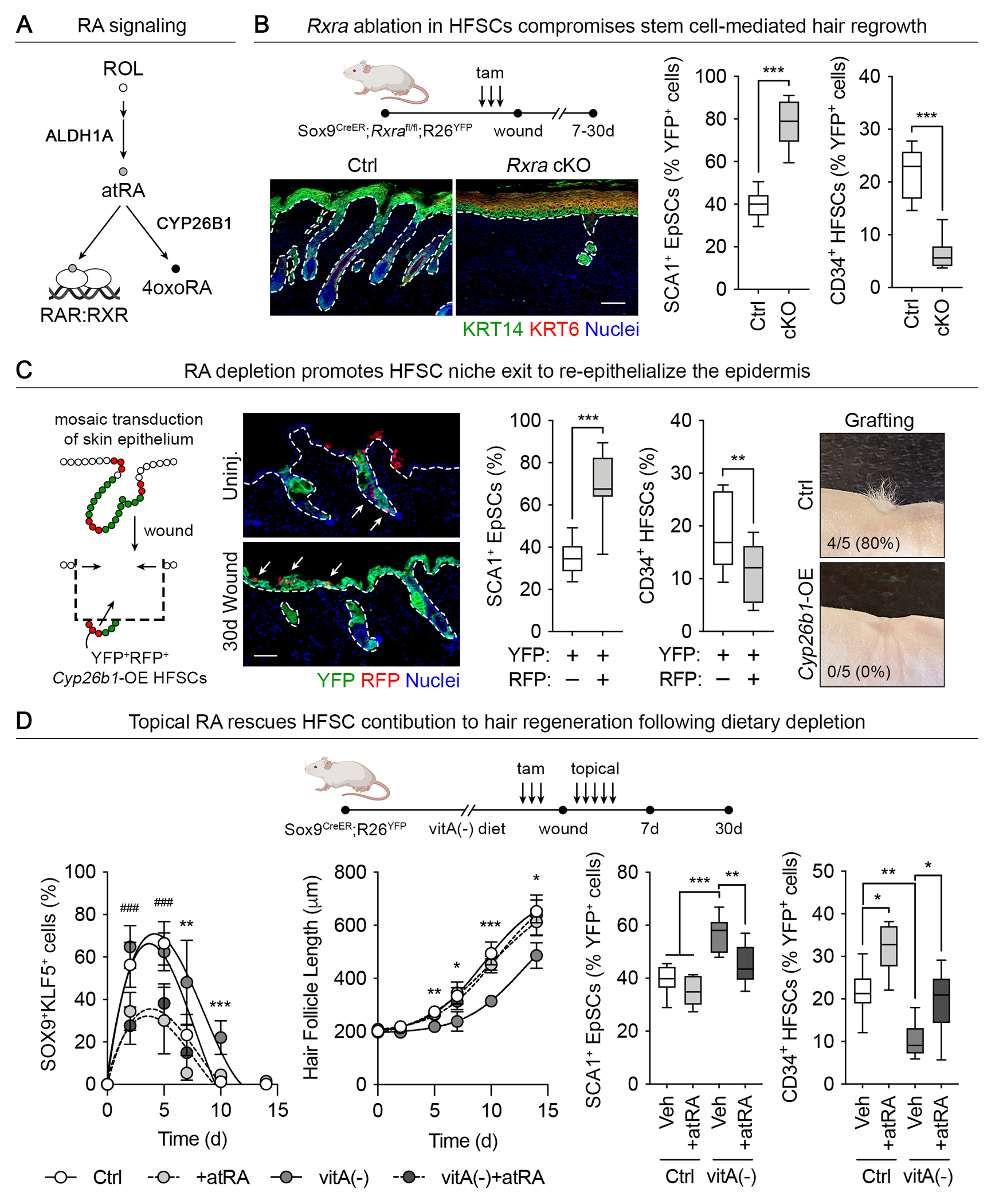
atRA/RAR/RXR dynamics regulate stem cell fate switching during wound repair in vivo. (**A**) Scheme depicting the steps and critical enzymes involved in atRA synthesis, degradation, and atRA-RAR-RXR mediated transcriptional activity. (**B**) *Rxra* was conditionally ablated in telogen-phase HFSCs of *Sox9*-CreER^+^ mice before abrasion (shallow) wounding. Note markedly altered tissue repair with *Rxra* cKO 7 days post-injury, including prolonged KRT6 (epidermal hyperproliferation) and blocked hair regeneration (left). The epidermal-dermal interface is denoted by dashed lines. Scale bar, 50μm. Quantification of lineage-traced HFSCs that mobilized to the epidermis at the expense of retention in the HF after *Rxra* cKO after 30 days post-injury by flow cytometry (n=6 to 8). (**C**) Transduction of *Sox9CreER;Rosa26-LSL*-*YFP* adult skin epithelium with a transgene encoding the atRA-degrading protein CYP26B1 (constitutive H2B-RFP control; left). Tamoxifen treatment activated *Cyp26b1* and enabled YFP^+^ HFSC lineage tracing after wounding. RFP^+^, i.e. *Cyp26b1*-overexpressing cells are present in uninjured HFs and epidermis but become overrepresented in the repaired epidermis relative to the HF after wound repair (center left). The epidermal-dermal interface is denoted by dashed lines; arrows refer to YFP^+^RFP^+^ cells of HFSC origin that overexpress *Cyp26b1*. Scale bar, 50μm. Quantification by flow cytometry (center right; n=11). Sustained *Cyp26b1* also precluded hair formation in HFSC skin grafts (right; n=5). (**D**) Mice were fed a normal or vitamin A-deficient diet and wounded in telogen. After repair was complete, YFP^+^ HFSCs were assessed for their contribution to skin barrier restoration or HF regeneration. Topical atRA applied during the first week post-wound accelerated lineage plasticity resolution (left, n=10 to 12 HFs per mouse, n=5 mice) and skewed HFSC contribution to hair regeneration, as measured by flow cytometry (right, n=10–12 HFs per mouse, n=5 mice), whereas vitamin A loss slowed HF regrowth at intermediate stages of repair (center, n=9–12 HFs, n=5 mice). Hair regeneration and HFSC fate were rescued by topical atRA treatment. Veh., vehicle.

The recruitment of HFSCs to epidermal repair is a multistep process first involving the entry of HFSCs into lineage plasticity, then a subsequent fate switch from HFSCs to EpSCs, and finally the rebalancing of stem cell numbers across tissue compartments. Having established that atRA signaling is transiently lost after injury (Fig. 2A), we turned to addressing whether blocking atRA signaling would more readily enable HFSCs to adopt an epidermal fate. We first conditionally deleted *Rxra* in HFSCs in telogen and then initiated a shallow wound. The loss of the transcriptional effector of atRA preferentially mobilized lineage-traced HFSCs to re-epithelialize the denuded epidermis (Fig. 5B and fig. S13A). By contrast, hair regrowth was also severely compromised, which suggests that restoring atRA activity after an injury might be necessary to resolve lineage plasticity and reinstate the production of HF fates.

To corroborate these findings, we again turned to our *in utero* delivery system to mosaically transduce the skin epithelium of *Sox9CreER;Rosa26-LSL-YFP* mice with an RFP marker and a constitutive *Cyp26b1*, a monooxygenase that catalyzes the degradation of atRA to 4-oxo-RA (51) (Fig. 5A). As verified in HFSC cultures, atRA was significantly more potent than 4-oxo-RA or other vitamin A derivatives (fig. S13B). Consequently, destabilizing intrinsic atRA levels by *Cyp26b1* overexpression had deleterious effects on HFSC identity and behavior (fig. S13C). In vivo, RFP^+^*Cyp26b1*^+^ HFSCs were significantly more likely compared with their WT counterparts to contribute to epidermal repair at the expense of remaining within the HF (Fig. 5C and fig. S13, D and E). *Cyp26b1* overexpression also prevented hair production in grafted HFSCs, confirming that endogenous atRA is a potent determinant of stem cell fate in a wounding scenario (Fig. 5C). When topically administered immediately after wounding, atRA but not 4-oxo-RA caused a higher fraction of HFSCs to remain in the ‘bulge’ instead of undergoing a fate change to become EpSCs, whereas RAR inhibition had the opposite effect and slowed HF downgrowth (fig. S14). Altogether, these finding underscore the importance of atRA to HFSC identity and function.

Finally, we assessed the consequences of tipping the balance towards lineage plasticity in a more clinically relevant setting by placing mice on a vitamin A-deficient diet and monitored the wound repair process. Wound-mobilized HFSCs experiencing vitamin A deficiency entered a prolonged state of dual lineage potential, quantified by SOX9 and KLF5 expression in lineage-traced HFSCs (Fig. 5D and fig. S15A). By contrast, topical atRA resulted in fewer HFSCs entering the lineage plasticity state. This extended lineage plasticity likely arose from reduced substrate availability rather than an altered rate of conversion to atRA because vitamin A deficiency did not alter ALDH1A expression (fig. S15B).

The consequences of prolonged lineage plasticity were substantial, as vitamin A-deficient wounds exhibited a marked delay in hair regeneration accompanied by an enhanced contribution to repairing epidermis (Fig. 5D, fig. S15C). Depilation in vitamin A-deficient mice also displayed significantly slowed hair regrowth while favoring lineage-traced HFSCs to repopulate the epidermis (fig. S15D). Each of these features were rescued by topically applying atRA to the wound bed, underscoring the tissue-specific nature of the response (Fig. 5D). Paradoxically, despite the increased flux of HFSCs into the epidermis in vitamin A-deficient wounds, restoration of the skin barrier was still delayed (fig. S15E). Stem cell expansion during repair was unchanged, pointing to delayed lineage plasticity resolution as the root of this barrier reformation deficit and illuminating the need to tightly regulate this process during an injury response.

## Discussion

Vitamin A deficiency is a major health issue globally, and topical retinoids are often used in both clinical and cosmetic applications to rejuvenate the skin (66, 67). However, despite the profound influence of retinoids on skin biology, the field has lacked a clear picture as to how atRA directs stem cell behavior in homeostatic or wound-induced regenerative processes. Although topical atRA stimulates folliculogenesis in full-thickness wounds (68, 69), excessive atRA availability has also been reported to inhibit hair cycling and cause alopecia (50, 51, 70). Similarly, although atRA has long been known to promote proliferation and suppress epidermal differentiation, both positive and negative effects have been described during epidermal repair (71, 72). The field is further complicated by the fact that many non-epithelial cell types present in barrier organs also respond to retinoids (73–75).

Traditionally, stem cell culture has been viewed as an attractive approach to unravelling such pleiotropic effects. However, this has not been the case for hair biologists despite skin being the pioneering model for stem cell culture (76). Our findings exposed lineage plasticity as a major impediment to obtaining meaningful mechanistic insights in vitro on how stem cells select cell fates in response to local niche signals. In so doing, we discovered that atRA levels function at the helm in governing stem cell identities within the skin, which in turn is essential for the tasks that these stem cells will perform in homeostasis and wound repair.

Our findings were particularly interesting in that atRA-induced signaling has also been found to control hematopoietic stem cell (HSC) behavior. In the bone marrow, atRA appears to maintain HSC quiescence under duress (77). In the skin, atRA functions more prominently in resolving lineage plasticity, being required for lineage specification, whereas quiescence seems more the task of BMP signaling. Nonetheless, the finding that atRA signaling functions critically in maintaining stemness of both HFSCs and HSCs suggests a broader and more important role for this metabolite in stem cell biology than previously realized.

In our studies, we showed that RXR transcription factors directly target master regulators at the core of stem cell identity in the skin, activating HFSC genes while suppressing an epidermal fate. These chromatin dynamics are essential for BMPs to work cooperatively with atRAs in maintaining HFSC identity and quiescence and for HFSCs to respond to external signals that orchestrate lineage determination in hair regeneration. That lineage plasticity must be resolved before stem cells can respond effectively to different maintenance and lineage-defining cues explains, at least in part, why so little progress has been made over the years in recapitulating the hair integumentary system outside the body.

We found that this knowledge could also be seamlessly applied to damaged skin, as stem cells must first enter this plastic state to cross tissue compartments and participate in their regeneration (4). We provide evidence that to do so, atRA levels must fall. Our data also imply that once re-epithelialization is complete, HFSCs and EpSCs must cede this cell fate flexibility so they can return to their physiological function, a process requiring the restoration of atRA levels. If atRA levels are too high, HFSCs fail to enter lineage plasticity and cannot contribute to epidermal repair. If too low, prolonged lineage plasticity disproportionally promotes epidermal fate contribution at the expense of hair regeneration. In either case, the ability to repair wounds and restore the skin barrier is delayed but for different underlying reasons, explaining prior ambiguities with regards to the role of retinoids in wound repair.

Our findings provide insights into the importance of resolving lineage plasticity to properly regenerate tissue. They also confirm atRA as a powerful rheostat of stem cell fate during repair. This notion paves the way for future dissection of niche complexities in controlling the production of all HF fates uncomplicated by secondary effects, including their interactions with other niche components including neurons, immune cells, lymphatics, melanocytes, and mesenchymal cells. Our platform will also be useful for the high-throughput examination of therapeutic modalities designed to treat skin and hair disorders, speeding the translation of basic research into clinical practice. Finally, given the broad effects of retinoids and the widespread occurrence of lineage plasticity in wounding and in cancer, the principles that we have established will likely be applicable to other conditions and tissues.

## Materials and Methods

### Mice and procedures

Mice were maintained and all animal experiments performed in the Association for Assessment and Accreditation of Laboratory Animal Care (AAALAC)-accredited Comparative Bioscience Center at The Rockefeller University (RU) in accordance with NIH guidelines for Animal Care and Use. All protocols were approved by the Institutional Animal Care and Use Committee (IACUC; protocols no. 23008-H and no. 23038-H) and RU’s Institutional Animal Care and Use Committee. The following mice were used and cross-bred as needed in this study, purchased or previously generated in the Fuchs laboratory as noted: Sox9-CreER (C57BL/6 background; donated by H. Akiyama) (10), Rxra;fl-fl (C57BL/6 background; JAX no. 013086) (78), Rosa26-YFP (C57BL/6J background; JAX no. 006148), Lgr5-EGFP-IRES-CreER (C57BL/6 background; JAX no. 008875), immune-incompetent nude (Nu/Nu, CRL no. 088), wild-type CD1/ICR (CRL no. 022), and C57BL/6J (JAX no. 000664). Mice of all strains were housed in an environment with controlled temperature and humidity on 12-hour light-dark cycles. Age- and sex-matched mice, including littermates whenever possible, were used. Tamoxifen (50 μg/g) was administered intraperitoneally to mice 1 to 2 weeks before to CreER-mediated lineage tracing experiments. EdU (25 μg/g) was intraperitoneally injected 1 to 4 hours before sacrifice to label proliferating cells.

Dietary interventions were initiated in adult mice during 2^nd^ telogen (~postnatal day 50 to 55). Mice were placed on a modified sterile/irradiated vitamin A-free diet (no. 5BTN; W.F. Fisher and Sons, Inc.) or maintained on a control diet (no. 5053; 15 Ul/g) for >3 months. Mice were anesthetized with isoflurane and given a preoperative subcutaneous injection of buprenorphine (0.1 mg/kg BW) every month for orbital sinus blood sample collection (0.5 cc) and 8 mm posterior skin biopsy collection to determine the levels of systemic and local retinol and retinyl esters by liquid chromatography-mass spectrometry (LC-MS). Animals were monitored daily for potential adverse health effects of dietary intervention and body weight was measured weekly.

Abrasion (shallow) wound experiments were performed using a method modified from Levy et al. (79). Adult mice during telogen were anesthetized with isoflurane and preoperative injection of buprenorphine (0.1 mg/kg BW) was administered subcutaneously. Back skins were shaven, treated with hair removal cream (Veet), and washed with sterile distilled water. Bare skins were gently stretched and a Dremel tool (100 series) with polishing wheel attachment (520; Dremel, Inc.) was used to remove the epidermis and upper HF by polishing a ~1×1cm portion of skin laterally 6–8 times. For depilation experiments, molten wax was applied to the hair coats of anesthetized mice and stripped after hardening. To assess barrier reformation, a Tewameter TM300 (Courage + Khazaka Electronic GmbH) was used to take transepidermal water loss (TEWL) measurements from the back skin of wounded mice. When measuring wounds that have not yet healed, the probe was pressed gently against the scab.

For all topical treatments during wound healing or in healthy skin and at the indicated times during the hair cycle, mice were anesthetized with isoflurane. 50 μl/cm^2^ (0.5 mg/cm^2^) atRA, AGN193109 (pan-RAR inhibitor), or vehicle (ethanol) control were applied topically daily for the indicated intervals. To confirm the hair cycle stage before treatment, back skin hair was trimmed during the prior telogen stage and monitored daily for hair regrowth using the guidelines provided by Muller-Rover *et al.* (80).

Chamber grafts using cultured HFSCs and freshly isolated dermal fibroblasts were performed as described in Weinberg *et al.* (56). Briefly, immuno-incompetent recipient nude mice were anesthetized using Ketamine (100 mg/kg BW)/Xylazine (10 mg/kg BW) before grafting and placed in autoclaved cage units for the duration of the experiment. Cells were mixed and transferred into a silicone dome (chamber) implanted onto the dorsal surface of the recipient mouse in a 1 cm skin graft bed. Chambers were removed 10 days post-implantation and the graft site was examined and photographed weekly for the appearance of skin and hair. Grafted skin was collected after 4–6 weeks and prepared for further examination using histological methods.

### In utero lentiviral transduction

*RARE* activity measurements (31, 32) and inducible *Cyp26b1* overexpression were achieved by in utero injection of lentivirus into the amniotic sacs of embryonic day (E) 9.5 mice, as previously described (27). The surface ectoderm exists as a single layer of unspecified progenitors at E9.5, which can be stably transduced without infecting dermal cell types or otherwise altering the embryonic environment. In brief, the *RARE*-RFP sequence (Addgene; plasmid #183054) was cloned alongside a ubiquitous phosphoglycerate kinase promoter (*Pgk*)-driven *H2B-GFP* to identify transduced cells. The full-length *Cyp26b1* open reading frame (Origene Technologies; NM_175475) was cloned downstream of a synthetic CAG promoter (frequently used to drive expression of mammalian transgenes), lox-stop-lox (LSL) sequence, and *Pgk*-*H2B-RFP*. After sequence verification and viral production, 1μl of either lentivirus was injected *in utero* into E9.5 wild-type embryos (*RARE*-RFP) or embryos carrying *Sox9-CreER* and *Rosa26-YFP* transgenes (*Cyp26b1*-OE). *CreER*, *H2B-GFP*, and *H2B-RFP* expression was validated in transduced pups after birth by quantitative polymerase chain reaction (qPCR) as needed. In this system, delivered cargo is stably integrated into the skin epithelial chromosomal DNA and propagated into adulthood in the epidermis and hair follicles. Lentiviruses can be titrated as desired to achieve either broader coverage (*RARE*-RFP) or mosaic infection to allow for direct comparison between infected and non-infected cells (*Cyp26b1*-OE).

### Liquid chromatography-mass spectrometry (LC-MS)

To quantify the impact of a vitamin A-deficient diet for 3 months on adult *Sox9-CreER;Rosa26*-*YFP* mice, serum and skin biopsies were stored @ −80°C until processing. Serum was resuspended in 0.1ml 75:25 acetonitrile:methanol extraction buffer containing 0.01% w/v butylated hydroxytoluene with 60nM D_6_-retinol and 10nM ^13^C_3_-retinyl palmitate (Cambridge Isotope Laboratories). Biopsies were resuspended in 0.5ml extraction buffer with 2.5nM D_6_-retinol and 0.5nM ^13^C_3_-retinyl palmitate and 5 mm bead-homogenized 2x at 30 sec^−1^ for 30 sec each using a TissueLyser II (Qiagen). After centrifugation for 10 min at 10,000 g, all supernatant was collected.

Extracts were reconstituted in 50 μl of 65:30:5 acetonitrile:isopropanol:water (v/v/v), vortexed for 15 sec, and centrifuged at 20,000 g for 30 min at 4°C. 5 μl of supernatant was injected into the LC-MS in a randomized order. Lipids were separated on an Ascentis Express C18 2.1 mm × 150 mm × 2.7 μm particle size column (Supelco) connected to a Vanquish UPLC system and a Q Exactive benchtop orbitrap mass spectrometer (Thermo Fisher), equipped with a heated electrospray ionization (HESI) probe (81). The mass spectrometer was operated with the following parameters: positive ion polarity; spray voltage, 3500 V; heated capillary temperature, 320 °C; source temperature, 250 °C; S-Lens RF level, 45; sheath gas, 60 (arbitrary units); auxiliary gas, 20 (arbitrary units). External mass calibration was performed every three days using the standard calibration mixture.

Mass spectra were acquired in both positive ionization modes using full MS and parallel reaction monitoring (PRM) scans. The full MS scan was acquired at 70,000 resolution, an AGC target of 1 × 10^6^, 200 ms max injection time, and a scan range of 200 to 700 m/z. The PRM scans were acquired at 17,500 resolution, an automated gain control (AGC) target of 1 × 10^5^, 50 ms max injection time, 1.6 Da isolation width, and a normalized collision energy (NCE) of 35 units. The inclusion list contained three-time dependent transitions: 0 to 30min, 269.2264 m/z (retinol/retinyl esters); 5 to 10 min, 275.2640 (D_6_-retinol); 15 to 25 min, 272.2364 (^13^C_3_-retinyl palmitate). Extracted ion chromatograms for retinol and retinol palmitate were integrated using Skyline Daily v22 (82) (MacCoss Lab) and a 2 parts per million (ppm) mass tolerance. The concentration was determined from the slope of calibration curves prepared by spiking in known concentrations of ^13^C_3_-retinyl-palmitate and D_6_-retinol into the corresponding biological matrix. Calibration curve data points were processed using the same workflow as the biological samples.

### FACS and analysis

To isolate *in vivo* epidermal, hair follicle, and wounded stem cell populations, mouse back skin was dissected and subjected to chemical and mechanical digestion. Telogen samples were scraped with a dull scalpel to remove excess fat and placed dermis side down in 0.25% trypsin-Ethylenediaminetetraacetic acid (EDTA, Gibco) for 35 min at 37°C. Wounded skin at early or intermediate stages of repair was placed in 0.25% trypsin-EDTA for 20 min, the eschar removed and scab scraped gently, and returned to incubation for 15 min. Anagen samples were subjected to 0.25% collagenase (Sigma) in Hank’s balanced salt solution (HBSS) for 35 min before 0.25% trypsin-EDTA for an additional 15 min. After digestion, single cell suspensions were obtained by scraping the epidermal side gently using a blunt scalpel. After washing with FACS buffer (5% FBS in phosphate buffered saline, PBS), cells were filtered through 70- and 45-μm strainers, pelleted, and washed once with FACS buffer. Cells were incubated with the appropriate antibodies listed in Table S2 in FACS buffer for 20 min on ice and washed with FACS buffer. Sorting was performed on a BD FACSAria II equipped with Diva software (BD Biosciences).

For flow cytometry analysis, live cell suspensions were collected and analyzed in the same way as sorting. For cell cycle quantification, EdU was administrated through intraperitoneal injection (0.025 mg/g body weight) 12 hr before euthanasia. Single cell suspensions were fixed in 4% paraformaldehyde (PFA) (Electron Microscopy Sciences), stained with cell surface antibodies as above, and permeabilized in 0.3% Triton X-100 in PBS (Sigma). EdU^+^ cells were fluorescently labeled using Click-iT chemistry and counterstained by FxCycle Violet (1:1,000; Invitrogen). Analyses were performed on a BD LSRFortessa (BD Biosciences). Quantification and plot generation to illustrate gating strategies were performed in FlowJo.

### Cell culture

HFSCs were collected for culture from the following mouse crosses: wild-type, *L*gr5-EGFP-IRES-CreER, Sox9-CreER;Rosa26-YFP, and Sox9-CreER;Rxra-fl/fl;Rosa26-YFP. The following HFSC lines were produced from lentiviral infection of wild-type cells: *Klf5-EGFP*, *Cxcl14-EGFP*, and *Cux1(IRS)* and *Cux1(HS)* reporter lines. Each reporter construct contained the *Pgk-RFP-NLS* transduction control for FACS purification post-infection. Both sexes were used for cell line derivation.

HFSC lines were established in 2D cultures, plated on mitomycin-inactivated feeders in E intermediate calcium (300 μM) medium (E300+Y) (83) supplemented with penicillin/streptomycin and 10 μM Y-27632 (Selleckchem). Cultures were incubated at 37°C and 7.5% CO_2_. Media was changed every 2 to 3 days. When near-confluent, HFSCs were digested with 0.25% trypsin-EDTA in PBS (Gibco) for 10 min at 37°C and resuspended with culture media for passaging. Only HFSCs propagated for ≤ 10 passages were used for downstream experiments.

For 3D cultures, dissociated HFSCs were resuspended at a density of 50 to 300 cells/μl in 40–75 μl 60% Cultrex BME (R&D Systems) droplets under low-attachment conditions (Nuclon Sphera, Thermo Scientific). After polymerization, cell droplets were initially grown in E300+Y medium supplemented with 50 ng/ml EGF or 10 ng/ml FGF10 (Peprotech) for 2 to 3 days to maximize colony forming efficiency and outgrowth. To resolve lineage plasticity, serum-replete culture media was washed with sterile PBS and changed to Dulbecco’s Modified Eagle Media/Ham’s F12 media (DMEM/F12) (3:1) containing 1x B-27 supplement (1:50; Gibco) (84), 125 mg/l cholesterol-cyclodextrin (Sigma), 300 μM calcium chloride, 10 μM Y-27632, 100 nM atRA (Sigma), and 1 μM Go6976 (PKCi; R&D Systems). Media was exchanged every two days for up to 14 days, as indicated.

To distinguish the impact of various media components on lineage plasticity, DMEM/F12 base medium was replaced by human plasma-like medium (HPLM, Gibco) and FBS was replaced by a cocktail of 5% BSA-conjugated palmitate, oleate, and linoleate (Sigma) with or without the following additives: 0.01 mM ethanolamine, 0.5 mM L-carnitine, 0.1 mM glutathione, 5 ng/ml sodium selenite, and 1 μM a-tocopherol (Sigma). All growth factors and small molecules tested for their effect on lineage plasticity and HFSC fate are listed in Table S1.

Daily brightfield imaging of HFSCs throughout colony formation and growth was performed using a BioTek Cytation5 multimodal reader (Agilent, Inc.). Before harvest, cells were washed with sterile PBS and directly fixed in 4% paraformaldehyde at 4°C, dissociated into a single cell suspension in TrpLE-Express (Gibco) for 15min at 37°C, or isolated from Cultrex as intact colonies using Cell Recovery Solution (Corning). To assess cell proliferation, cells were incubated with 10μM EdU for 4–12 hr prior to trypsinization and fixation.

### ATAC-seq

ATAC-seq was performed on 100,000 cultured HFSCs for each condition and replicate as previously described (85). Briefly, cells were lysed for 5 min and transposed with Tn5 transposase (Illumina) for 30 min. Samples were barcoded and sequencing libraries were prepared according to the manufacturer’s guidelines (Illumina) and sequenced on an Illumina Novaseq 6000. For sequencing analysis, 50 base pair (bp) paired-end FASTQs were aligned to the mouse genome (GRCm38/mm10) using the Bowtie (v.1.1.2) (86). Aligned reads were de-duplicated with Picard (v.2.3.0; Broad Institute, 2019) and shifted to correct for Tn5 insertion bias. Peaks were called using MACS2 (v.2.1.1) with default settings (87). Next, all peaks from each condition were concatenated to a union peak set and the read coverage of each sample at these peaks was calculated with Bedtools (v.2.25). The R (v.4.2.3) package edgeR (v.3.40.2) (88) was used for differential peak analysis and use for principle component analysis (PCA). pheatmap (v.1.0.12) was then used to generate the heatmap and generating the K-means clusters in R. For motif analysis, HOMER (v.4.11) findMotifGenome.pl was used (89).

### RNA-seq

Total RNA from 100,000 HFSCs for each sample was collected in Trizol and purified using the Direct-zol RNA MiniPrep kit (Zymo Research) according to the manufacturer's instructions. Deoxyribonuclease (DNase) treatment was performed to remove genomic DNA (RNase-Free DNase Set, Qiagen). Sample quality was determined using an Agilent 2100 Bioanalyzer; all samples for sequencing had RNA integrity numbers (RIN) >8. cDNA library construction was performed by the Rockefeller University (RU) Genomic Core facility and cDNA libraries were sequenced on an Illumina Novaseq 6000. Transcript abundance quantified using Salmon (v.1.4.0) with a reference genome sequence fetched from BSGenome.Mmusculus.UCSC.mm10 package. Gene level counts and transcripts per million (TPM) were calculated using Tximport (v.1.12.3) package. DEGs from raw counts were generated with DESeq2 (v.1.16.1). pheatmap (v.1.0.12) was used to generate the heatmap and generating the K-means clusters in R (v.4.2.3).

### CUT&RUN (CNR)

500,000 cultured HFSCs for each sample were collected and CNR was performed as previously described (90) with minor modifications. Cells were washed with ice-cold PBS, resuspended in crosslinking buffer (10 mM HEPES–NaOH pH 7.5, 100 mM NaCl, 1 mM egtazic acid (EGTA), 1 mM EDTA and 1% formaldehyde) and rotated at room temperature for 10 min. Crosslinked cells were quenched with glycine at a final concentration of 0.125 M for 5 min at RT, washed with cold 1× PBS, and resuspended in NE1 buffer (20 mM HEPES–KOH pH 7.9, 10 mM KCl, 1 mM MgCl_2_, 1 mM dithiothreitol, 0.1% Triton X-100 supplemented with Roche complete protease inhibitor EDTA-free) and rotated for 10 min at 4°C. Nuclei were washed twice with CNR wash buffer (20 mM HEPES pH 7.5, 150 mM NaCl, 0.5% bovine serum albumin and 0.5 mM spermidine supplemented with protease inhibitor) and incubated with concanavalin-A (ConA) beads washed with CNR binding buffer (20 mM HEPES–KOH pH 7.9, 10 mM KCl, 1 mM CaCl_2_ and 1 mM MnCl_2_) for 10 min at 4°C. ConA-bead-bound nuclei were incubated overnight at 4°C in CNR antibody buffer (CNR wash buffer supplemented with 0.1% Triton X-100 and 2 mM EDTA) with the indicated antibody, washed once with CNR Triton wash buffer (CNR wash buffer supplemented with 0.1% Triton X-100), and incubated at 4 °C for 1 h in CNR antibody buffer and 2.5 μl pAG-MNase (EpiCypher). ConA-bound-nuclei were then washed twice with CNR Triton wash buffer, resuspended in 100 μl Triton wash buffer, and incubated on ice for 5 min. Next, 2 μl 100 mM CaCl_2_ was added and mixed gently to 100 μl ConA-bound-nuclei at 0°C for 30 min. The reaction was stopped by addition of 100 μl 2× stop buffer (340 mM NaCl, 20 mM EDTA, 4 mM egtazic acid, 0.1% Triton X-100 and 50 μg ml^−1^ RNaseA) and incubated at 37°C for 10 min. After incubation, ConA-bound-nuclei were captured using a magnet and supernatant containing CNR DNA fragments were collected. The supernatant was incubated at 70°C for 4 h with 2 μl 10% sodium dodecyl sulfate and 2.5 μL 20 mg ml^−1^ proteinase K. DNA was purified using PCI reagent (phenol:chloroform:isoamyl alcohol, Millipore) and overnight ethanol precipitation with glycogen at −20 °C. DNA was resuspended in elution buffer (1 mM Tris–HCl pH 8.0 and 0.1 mM EDTA). CNR sequencing libraries were generated using NEBNext Ultra II DNA Library Prep Kit for Illumina and NEBNext Multiplex Oligos for Illumina. PCR-amplified libraries were purified using 1× ratio of SPRI beads (Beckman) and eluted in 15 μl EB buffer (Qiagen). All CNR libraries were sequenced on an Illumina NextSeq using 40 bp paired-end reads. Reads were trimmed with Skewer, aligned to reference genome (mm10) using Bowtie (v.1.1.2), deduplicated with Java (v.2.3.0) Picard tools, and filtered to ≤120 bp using Samtools (v.1.3.1). BAM files for each replicate were combined using Samtools. Bigwig files were generated using Deeptools (v.3.1.2) with reads per kilobase of transcript per million mapped reads (RPKM) normalization and presented with Integrative Genomics Viewer (IGV) software. CNR peaks were called using SEACR (91) from bedGraph files generated from RPKM-normalized Bigwig files (UCSC Tools) using stringent setting and a numeric threshold of 0.001.

### scRNA-seq and analyses

In vivo skin and hair follicle cells were isolated from AnaVI (~postnatal day 32) back skin and enriched by FACS for CD45^−^CD31^−^CD140a^−^CD117^−^ cells as described. A subset of CD34^+^ HFSCs were simultaneously isolated from these mice and cultured for 2 passages before apportioning into 3D cultures using serum-replete media, serum-free media targeting lineage plasticity, and directed HF lineage progression and differentiation as described. Cultured HFSCs derived from the same line were labeled with TotalSeq-B hashtag antibodies (BioLegend) and multiplexed into single GEM wells. All cell types were resuspended at a final concentration of 1,200 cells/μl and sequenced on a NovaSeq 6000 (Illumina).

FASTQ generation, read alignment, filtering, hashtag oligonucleotide (HTO) classification, and count matrix generation were performed using CellRanger v7.0.0 pipelines and the GRCm38 (mm10) reference genome. Filtering low quality cells, empty droplets, and cells with high mitochondrial content following a standard pre-processing workflow in Seurat (58). Ambient RNA and doublet removal was performed on normalized and log-transformed data using SoupX (92) and DoubletFinder (93) computational tools, respectively. To factor out differences in cell cycle phase amongst proliferating cells, we scored and regressed out the difference between the S and G2/M phases.

Dimensional reduction, clustering, and visualization by UMAP was performed in Seurat. For the cultured HFSC subset before/after lineage plasticity resolution, cluster annotation relied upon differentially expressed features and separate analyses identifying gene signatures unique to HFSCs, EpSCs, and lineage-traced HFSCs during wound repair. Signatures were generated via bulk RNA-seq DEG via DESeq2 using the likelihood ratio test (LRT) (60) and data from Ge *et al.* (4), reduced to the top 50 most differentially expressed genes for each cell type.

In vivo HF cells were separately integrated with cultured HFSCs treated with atRA and PKCi following directed HF lineage progression and differentiation using Harmony (57). Marker gene expression profiles and differentially expressed features were used for cell type identification across clusters. The similarity of cultured HFSCs to in vivo HFs was separately queried by defining a set of anchors to produce cluster assignments and prediction scores against the in vivo reference dataset in Seurat. Single cell trajectories in cultured HFSCs were constructed using Monocle3 (59). After ordering cells in pseudotime, placing *Sox9*^+^ HFSCs as the root node, cell type-specific gene signatures identified by Yang *et al.* (46) were converted into module scores and used to infer each stage of stepwise HFSC differentiation. Finally, selected culture conditions and *in vivo* HF clusters were separated and grouped for pseudobulk DEG using DESeq2/LRT. Significant genes were defined by a log2-fold change > 1.0 and an adjusted p-value < 0.01.

### Immunofluorescence and microscopy

Tissue samples were fixed in 1–4% PFA immediately after dissection, dehydrated in 30% sucrose overnight at 4°C, embedded into OCT compound, and frozen. 14 to 20μm thick tissue sections were cut using a cryostat (Leica) and deposited onto SuperFrost Plus slides (VWR). Cultured HFSC colonies were resuspended and fixed in 1% PFA. All samples were blocked using 2.5% normal donkey serum, 1% BSA, 1% fish gelatin, and 0.3% Triton X-100 in PBS. Primary antibodies were applied in blocking buffer overnight, washed with 0.1% Triton X-100 in PBS, and then incubated with secondary antibodies and 1 μg/ml Hoechst 33342 (Thermo Fisher). All primary antibodies are listed in Table S2 and were used in combination with Alexa 488-, Alexa 594-, and Alexa 647-conjugated secondary antibodies (1:1,000; Life Technologies). For EdU incorporation, Click-iT EdU AlexaFluor imaging kits (Invitrogen) were used according to the manufacturer’s instructions. Samples were mounted using Fluoromount-G mounting medium (SouthernBiotech).

Epifluorescence microscopy was performed using an Axio Observer Z1 microscope equipped with a Hamamatsu ORCA-ER camera (Hamamatsu Photonics) and ApoTome.2 slider (Carl Zeiss) using 20x, 40x oil, or 63x oil objectives and Zen software. Confocal microscopy was performed on a Dragonfly spinning disk imaging system (Andor) with a Leica DMi8 Stand and cMOS Zyla camera using Andor IQ3 and Fusion software. All Z-stacked images (1 μm steps) were displayed as max intensity projections. Fluorescence intensity measurements were measured within nuclear masks generated in Fiji. 3D reconstructions of cultured HFSC colonies were generated in Imaris.

### Real-time qPCR

Cultured HFSCs were recovered from Cultrex using Cell Recovery Solution (Corning) and lysed in Trizol (Invitrogen). Total RNA was purified using RNeasy Micro or Mini kits (Qiagen) according to the manufacturer’s instructions. DNase treatment was performed on-column to remove genomic DNA. Equivalent amounts of RNA were reverse transcribed by Superscript VILO reverse transcriptase (Thermo Fisher). cDNAs were mixed with gene-specific primers listed in Table S3 and SYBR Green PCR Master Mix (Thermo Fisher) and quantified using the Applied Bio-systems QuantStudio 6 Real-Time PCR system. All cDNAs were normalized to equal amounts using primers against *Ppib2*.

### Western blotting

Recovered HFSCs were washed in cold PBS and lysed with Radioimmunoprecipitation assay (RIPA) buffer (Thermo Fisher) containing protease (Roche) and phosphatase inhibitors (PhosSTOP). Protein lysates were clarified by centrifugation and concentration was measured by BCA (Pierce). Lysates were denatured at 95°C for 5 min, separated by gel electrophoresis using a 4 to 12% NuPAGE Bis-Tris gradient (Life Technologies), and transferred to PVDF membranes (GE Healthcare, 0.45 μm). Membranes were blocked with Odyssey blocking buffer (Licor) and incubated with primary antibodies overnight. All primary antibodies are listed in Table S2. After washing with 0.1% Tween20 in PBS, membranes were incubated with IRDye 680/800 secondary antibodies (1:10,000, Licor), washed, and imaged with an Odyssey CLx Imager (Licor). Owing to multiple targeted proteins in each experiment, split samples with the identical sample volumes and processing procedures were blotted in parallel when necessary.

### Statistics and study design

For mouse experiments, sample sizes were selected by statistical power analysis whenever applicable (94). Animals were assigned randomly to experimental groups and studies were not blinded. All data from every experiment were included for analysis unless an error in experimental procedure was detected via failed positive and/or negative controls; in these cases, the whole experiment was excluded from analysis. Statistical and graphical data analyses were performed using Microsoft Excel and Prism9 (Graphpad) software. The number of replicates used in each experiment are indicated in figure legends. Data are presented as mean ± SEM (***P < 0.001, **P < 0.01, * *P* < 0.05) unless otherwise indicated. All statistical tests were two-sided unless not applicable.

## Supplementary Material

Supplemental Figure 1

Supplemental Figure 2

Supplemental Figure 3

Supplemental Figure 4

Supplemental Figure 5

Supplemental Figure 6

Supplemental Figure 7

Supplemental Figure 8

Supplementary Figure 9

Supplementary Figure 10

Supplementary Figure 11

Supplementary Figure 12

Supplementary Figure 13

Supplementary Figure 14

Supplementary Figure 15

Table S1

Table S2

Table S3
