# Supplementary figures and images for: Vitamin A resolves lineage plasticity to orchestrate stem cell lineage choices
Source: Science. Author manuscript; Available in PMC 2024 Jun 14. (PMC11177320; doi:10.1126/science.adi7342)

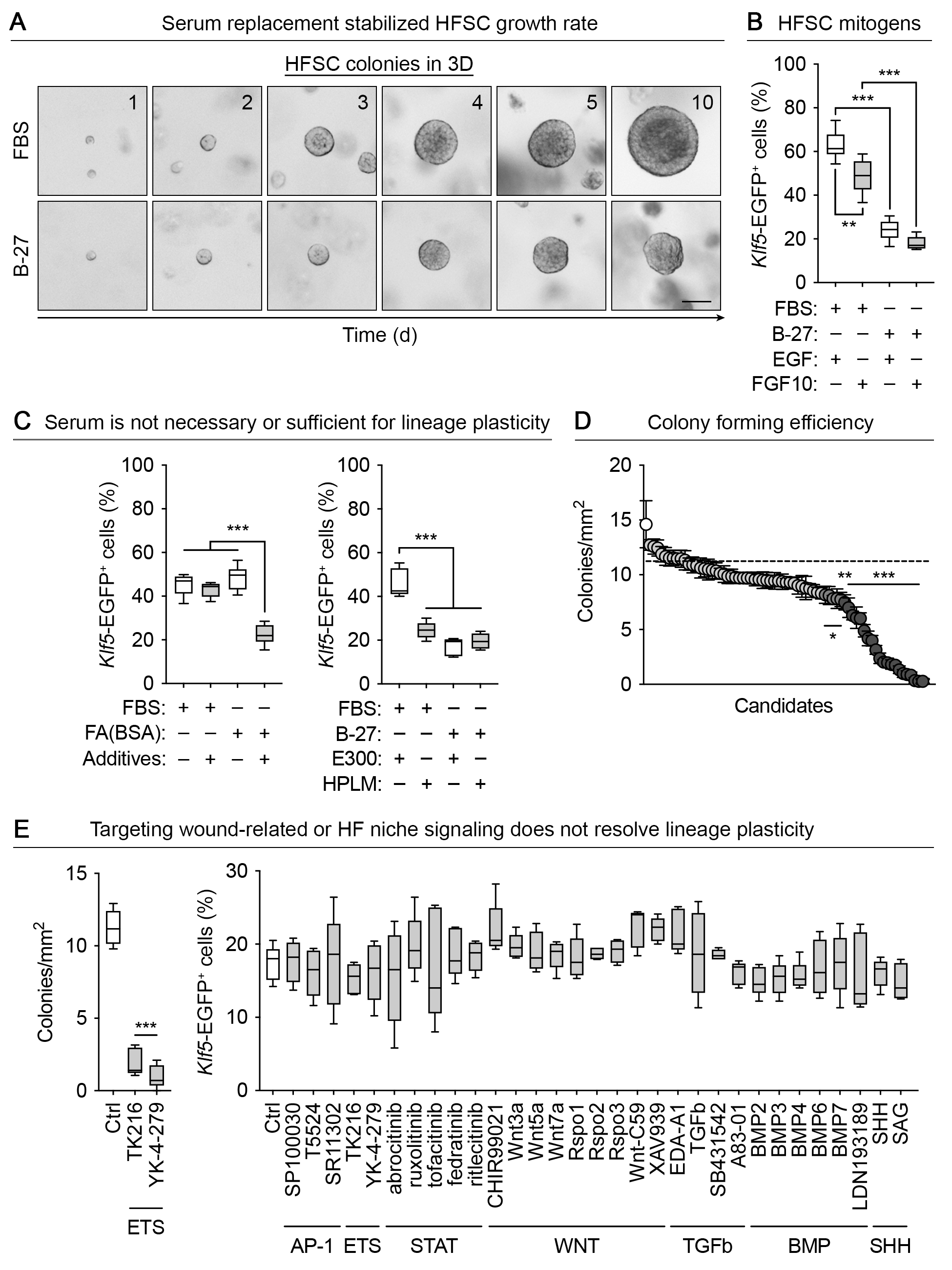

Supplement: Supplemental Figure 1 [file NIHMS1991969-supplement-Supplemental_Figure_1.tif]

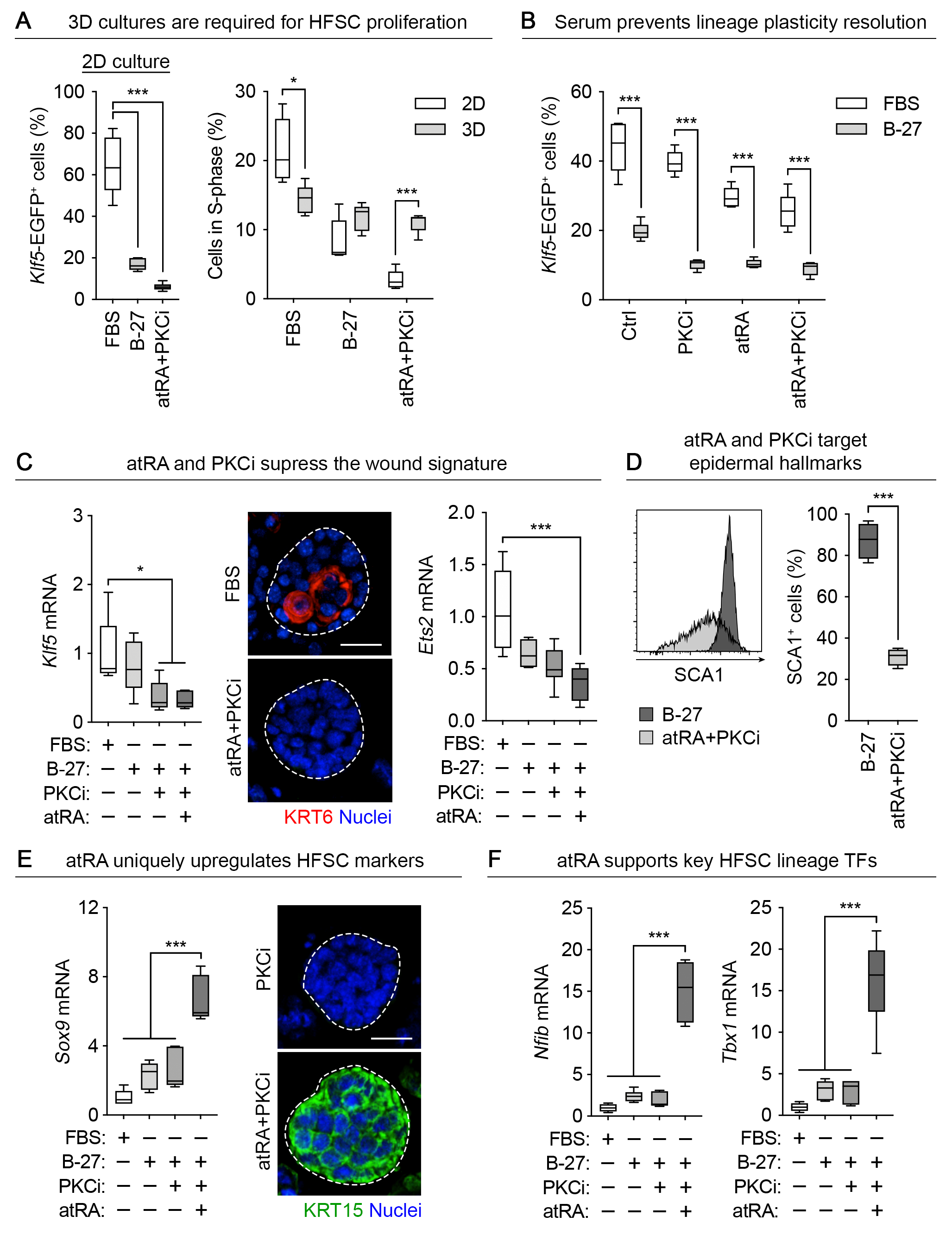

Supplement: Supplemental Figure 2 [file NIHMS1991969-supplement-Supplemental_Figure_2.tif]

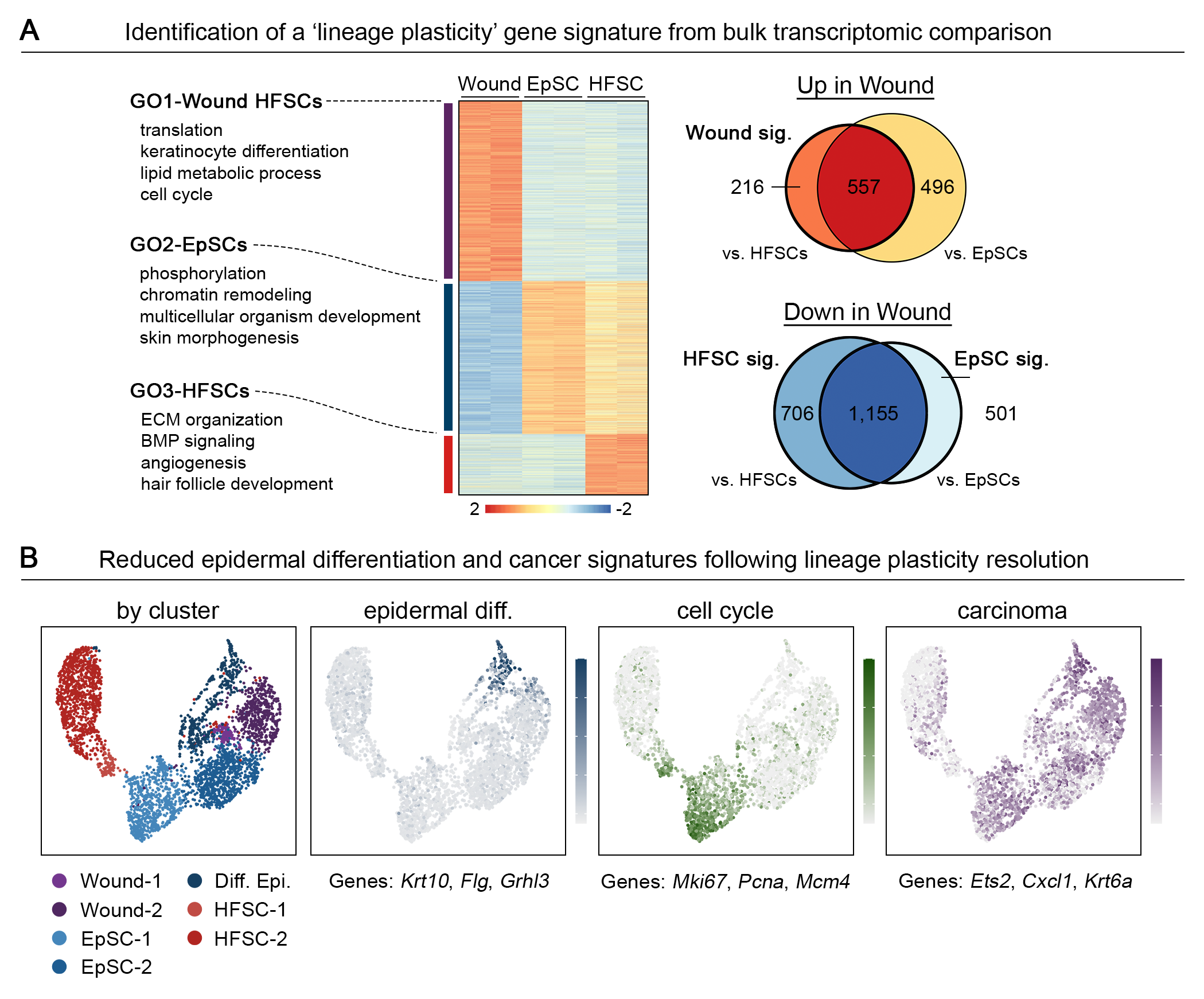

Supplement: Supplemental Figure 3 [file NIHMS1991969-supplement-Supplemental_Figure_3.tif]

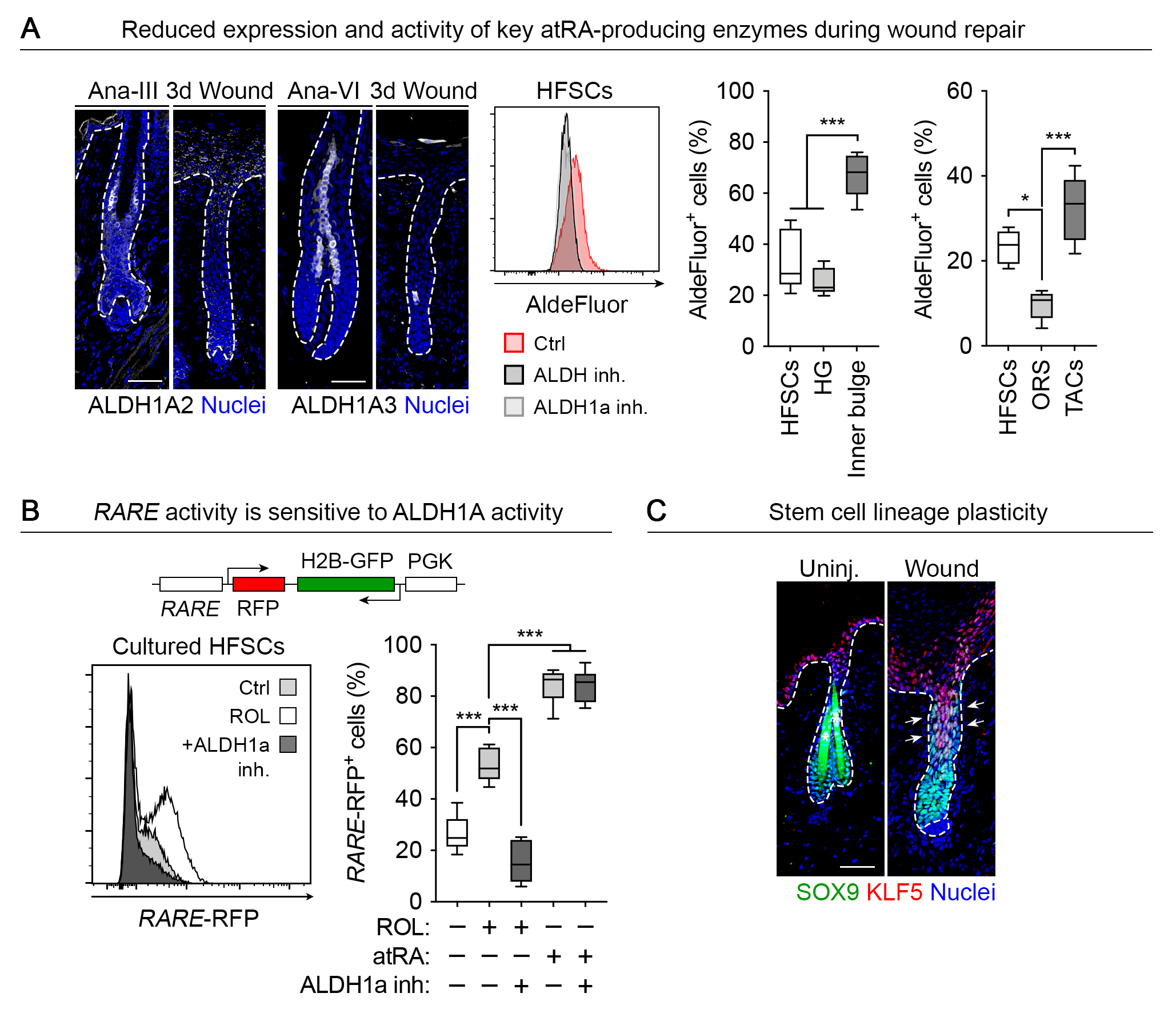

Supplement: Supplemental Figure 4 [file NIHMS1991969-supplement-Supplemental_Figure_4.tif]

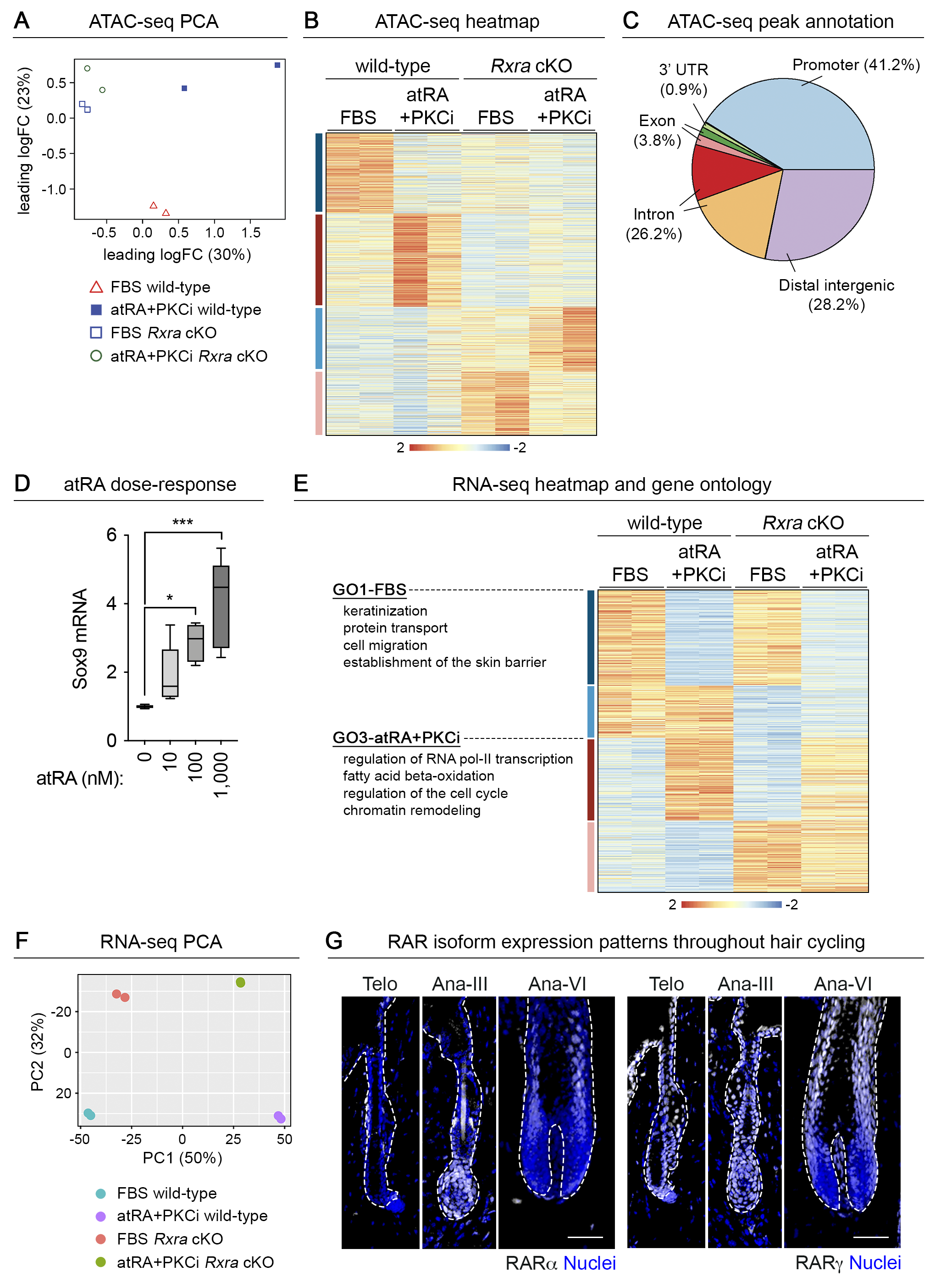

Supplement: Supplemental Figure 5 [file NIHMS1991969-supplement-Supplemental_Figure_5.tif]

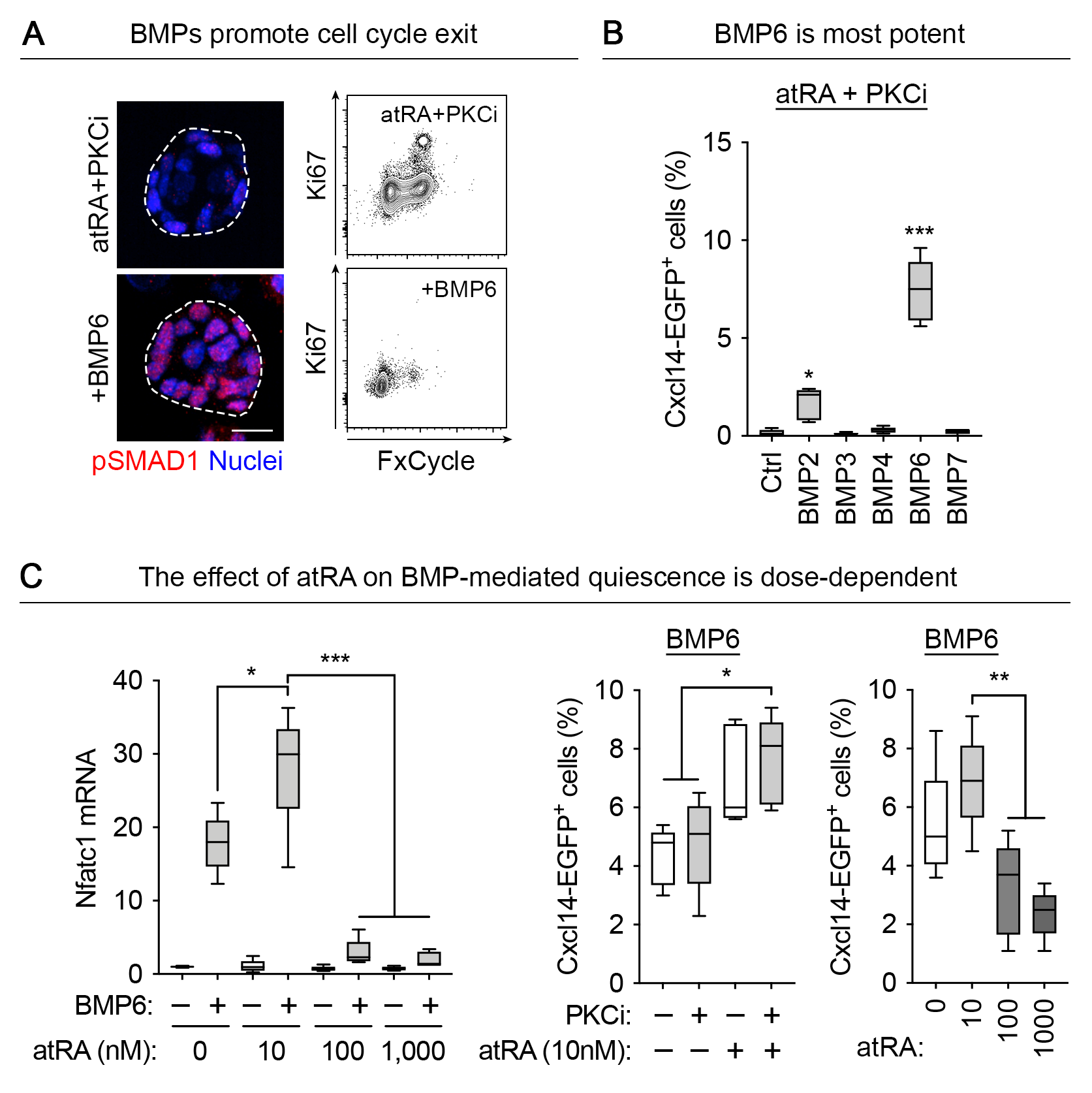

Supplement: Supplemental Figure 6 [file NIHMS1991969-supplement-Supplemental_Figure_6.tif]

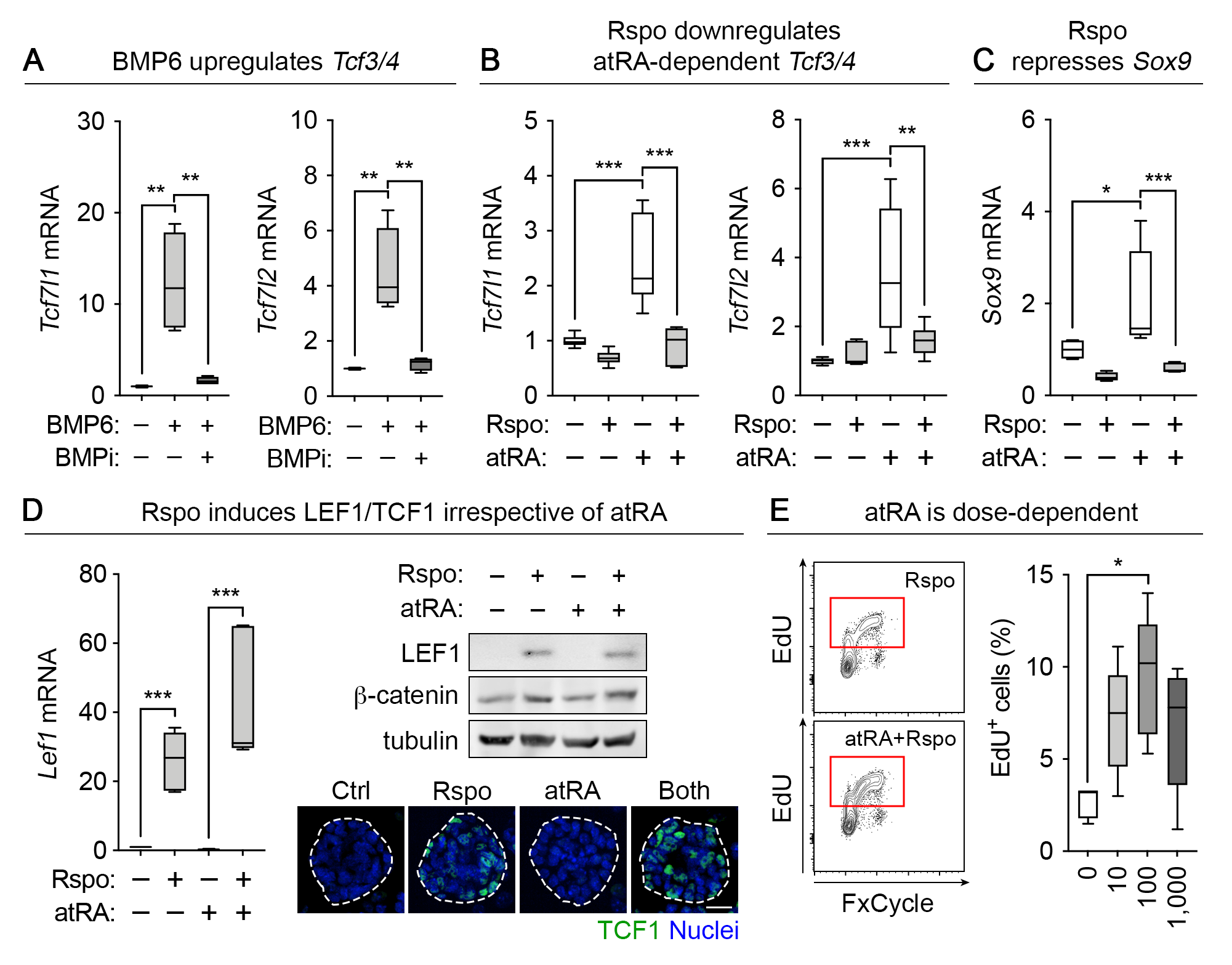

Supplement: Supplemental Figure 7 [file NIHMS1991969-supplement-Supplemental_Figure_7.tif]

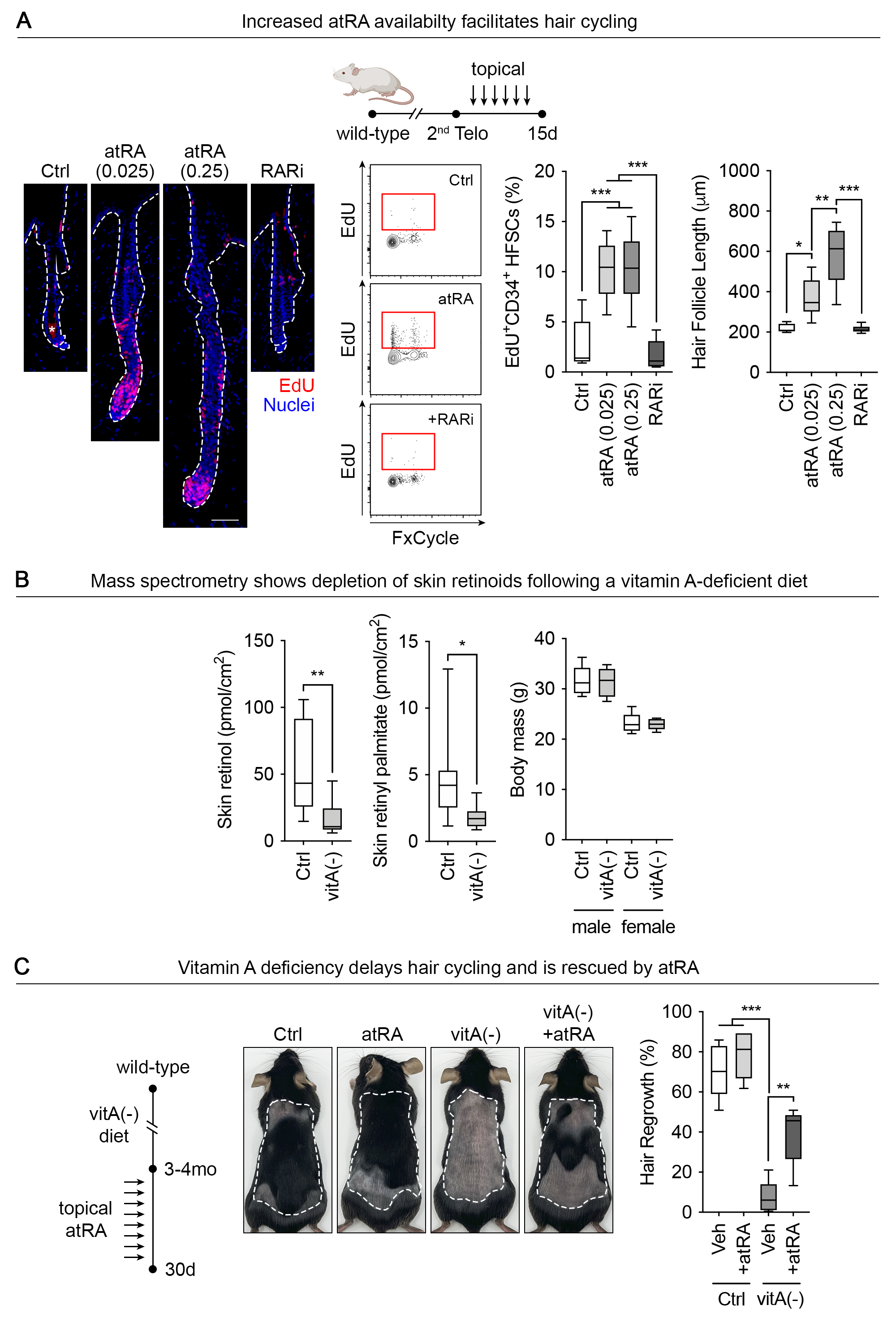

Supplement: Supplemental Figure 8 [file NIHMS1991969-supplement-Supplemental_Figure_8.tif]

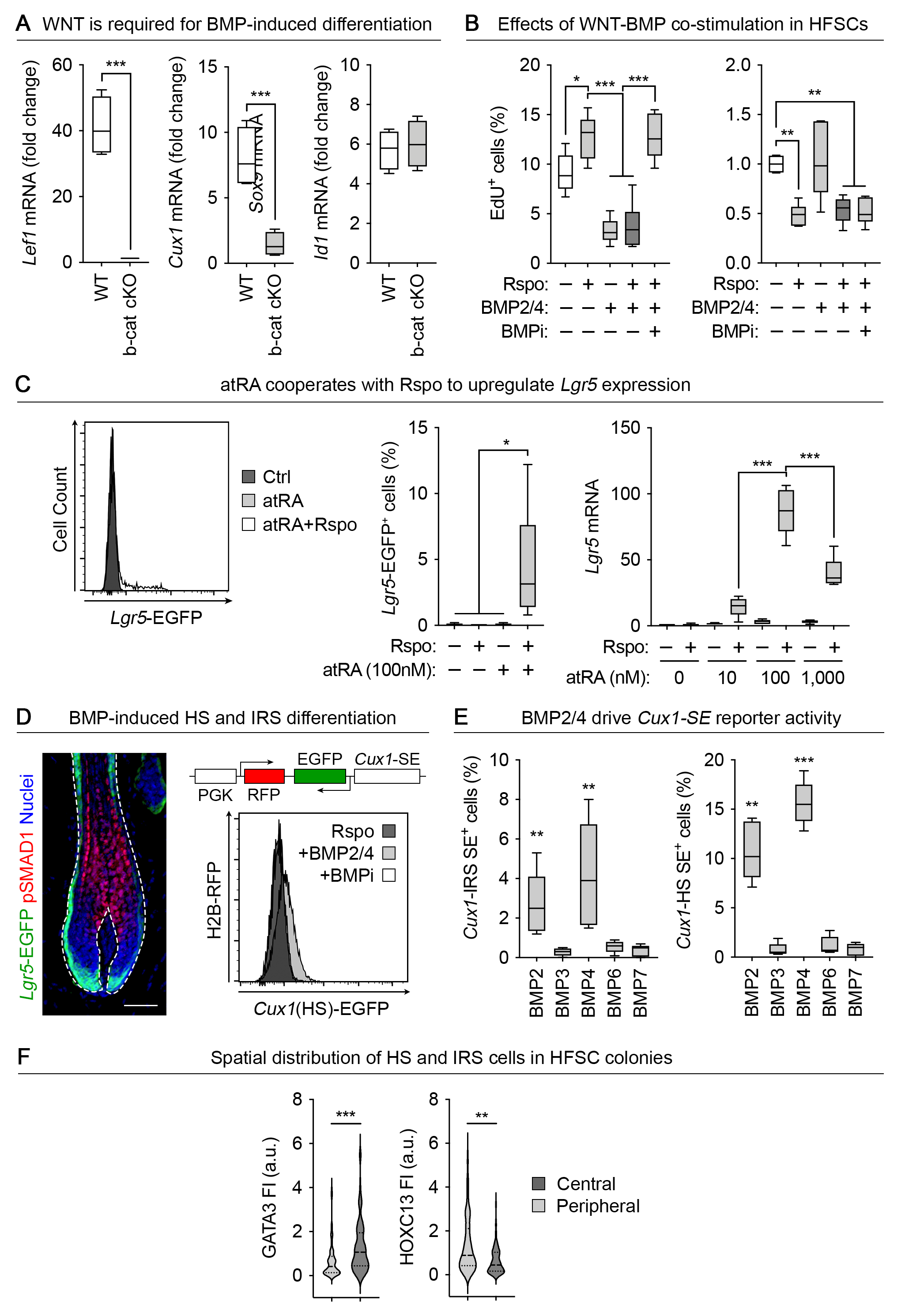

Supplement: Supplementary Figure 9 [file NIHMS1991969-supplement-Supplementary_Figure_9.tif]

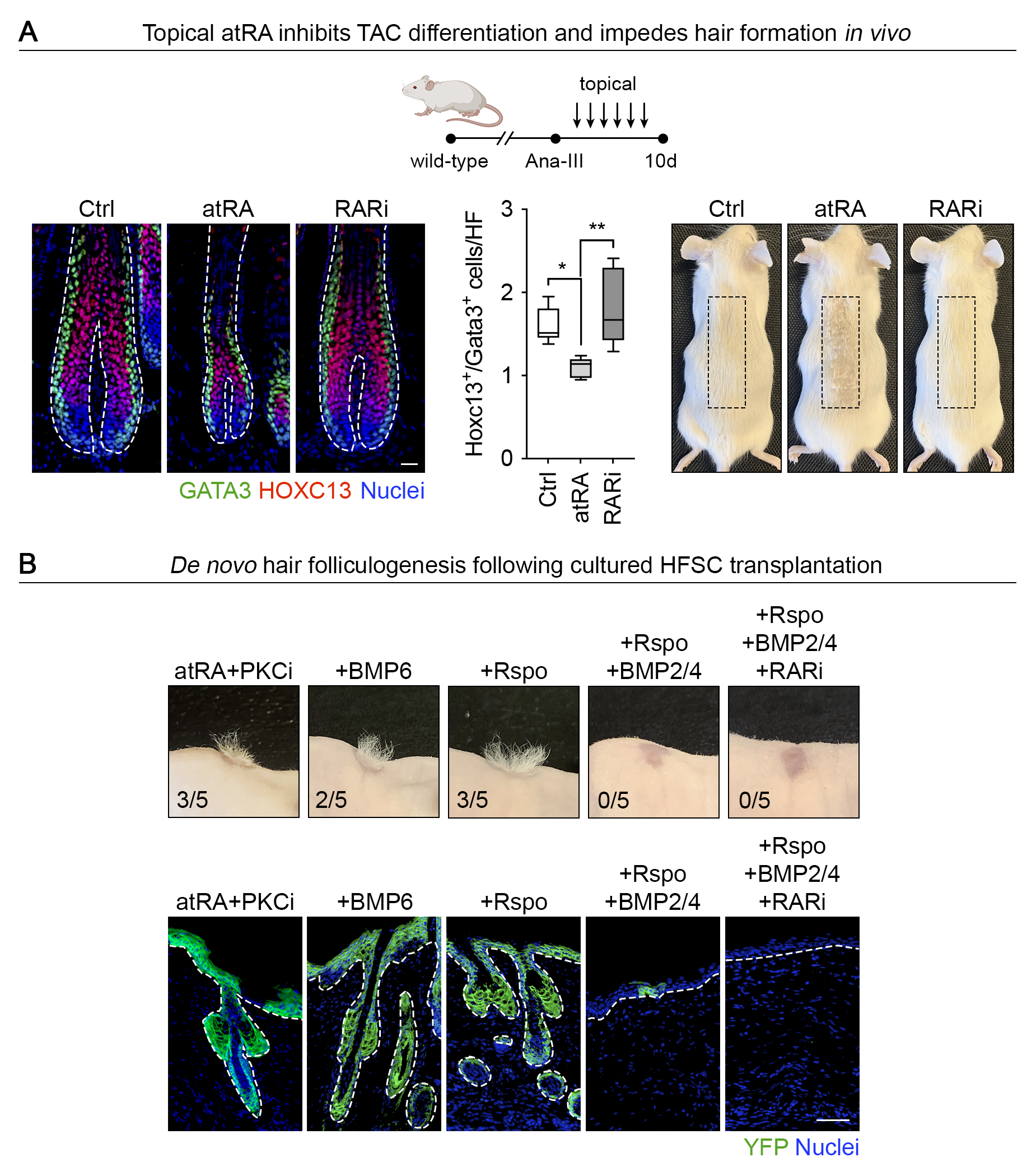

Supplement: Supplementary Figure 10 [file NIHMS1991969-supplement-Supplementary_Figure_10.tif]

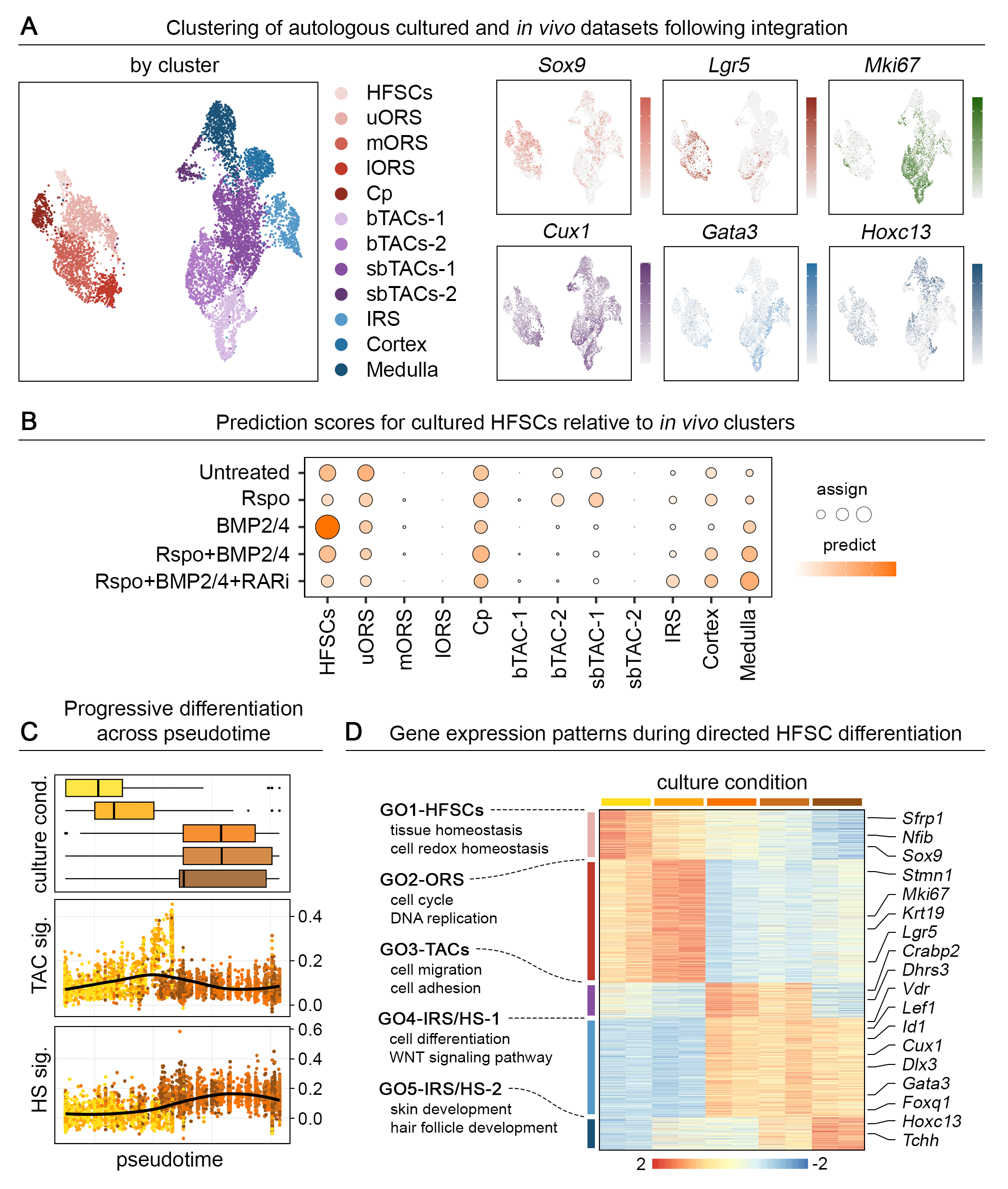

Supplement: Supplementary Figure 11 [file NIHMS1991969-supplement-Supplementary_Figure_11.tif]

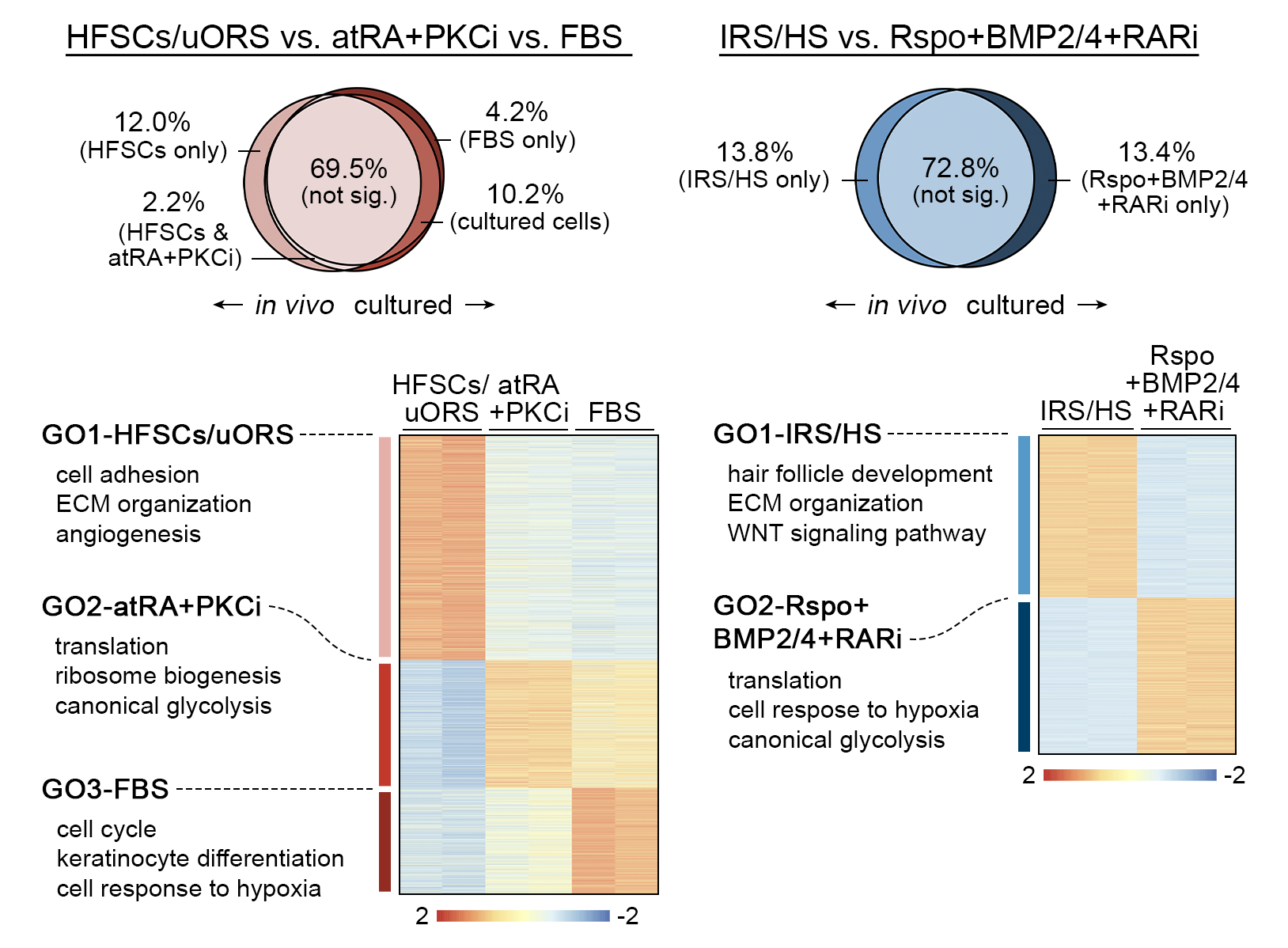

Supplement: Supplementary Figure 12 [file NIHMS1991969-supplement-Supplementary_Figure_12.tif]

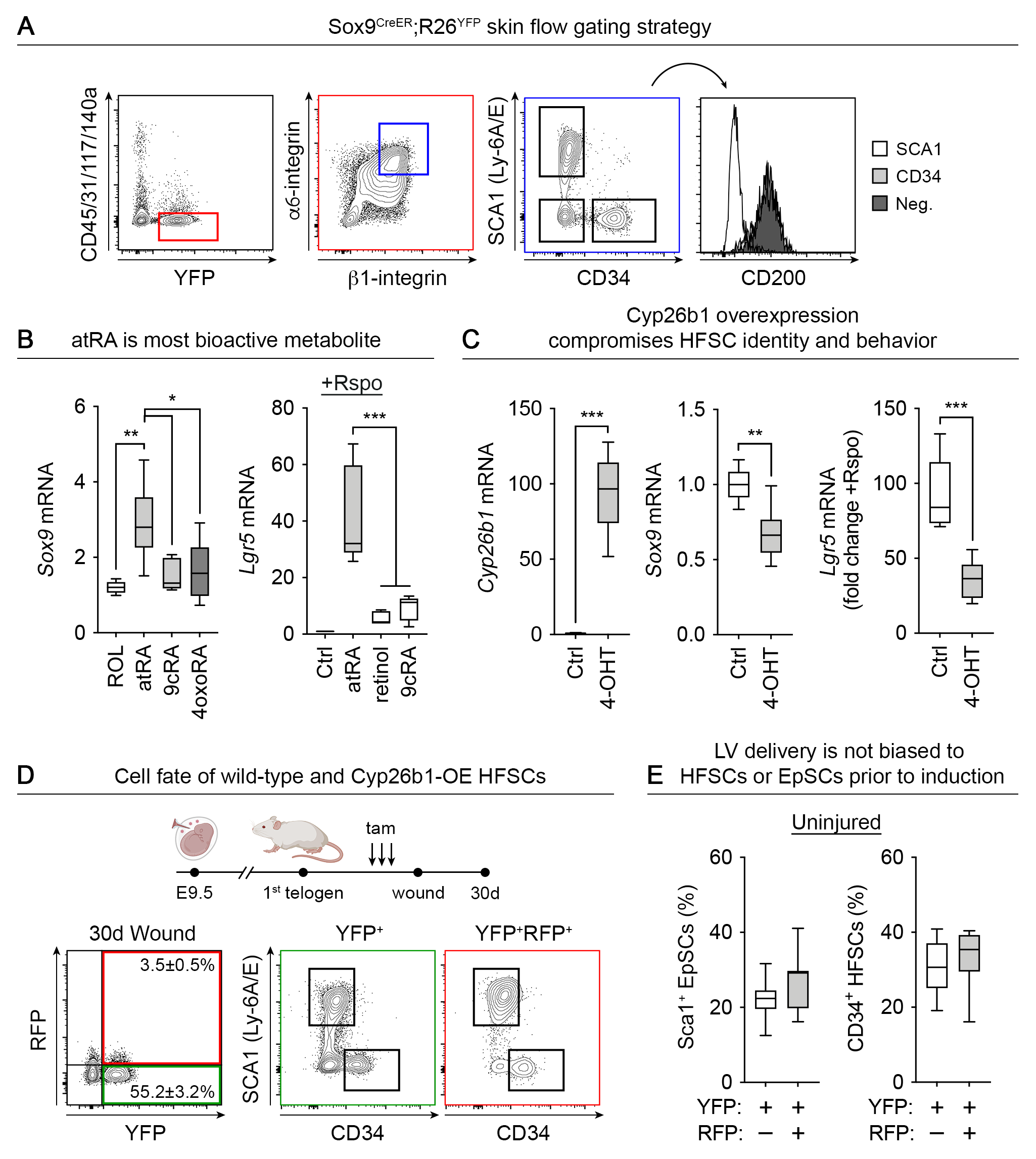

Supplement: Supplementary Figure 13 [file NIHMS1991969-supplement-Supplementary_Figure_13.tif]

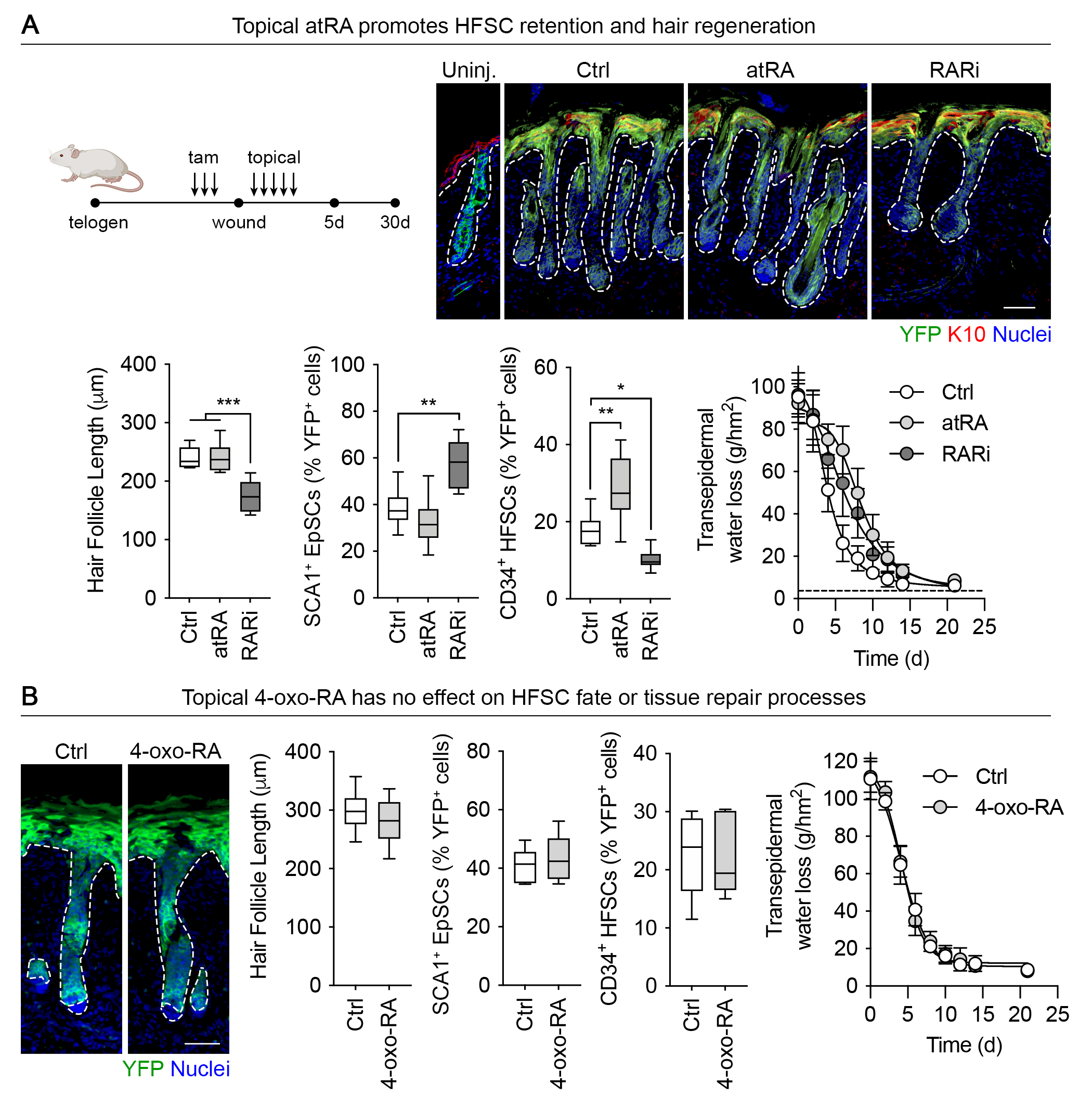

Supplement: Supplementary Figure 14 [file NIHMS1991969-supplement-Supplementary_Figure_14.tif]

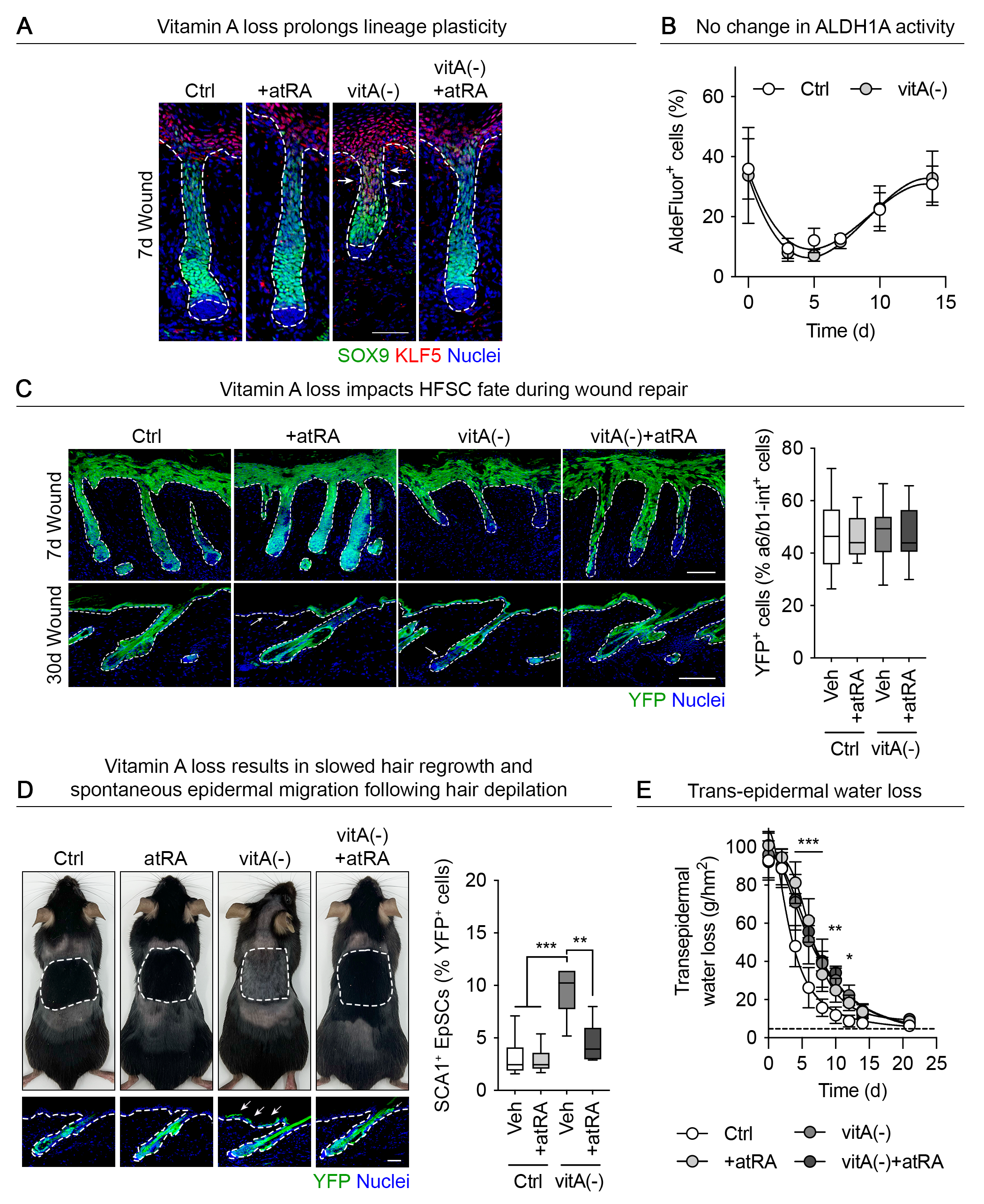

Supplement: Supplementary Figure 15 [file NIHMS1991969-supplement-Supplementary_Figure_15.tif]
