# Supplementary material for: Vitamin A resolves lineage plasticity to orchestrate stem cell lineage choices
Source: Science. Author manuscript; Available in PMC 2024 Jun 14. (PMC11177320; doi:10.1126/science.adi7342)
Supplement: Table S1 [file NIHMS1991969-supplement-Table_S1.pdf]

Small molecule/growth factor	Solvent	Working conc.	
10058-F4	DMSO	0.025	mM
17-AAG	DMSO	0.002	mM
9-cis-RA	DMSO	0.001	mM
A83-01	DMSO	0.001	mM
abrocitinib	DMSO	0.01	mM
AG490	DMSO	0.01	mM
AG879	DMSO	0.01	mM
AICAR	PBS	1	mM
Ascorbate	dH2O	0.025	mM
atRA	DMSO	0.001	mM
b-estradiol	DMSO	0.001	mM
BMP2	0.1% BSA in PBS	0.02	ug/ml
BMP3	0.1% BSA in PBS	0.02	ug/ml
BMP4	0.1% BSA in PBS	0.005	ug/ml
BMP6	0.1% BSA in PBS	0.02	ug/ml
BMP7	0.1% BSA in PBS	0.025	ug/ml
Calcipotriol	DMSO	0.00001	mM
Calcitriol	DMSO	0.00001	mM
CGP77675	DMSO	0.001	mM
CH223191	DMSO	0.001	mM
CHIR99021	DMSO	0.001	mM
CID 5951923	DMSO	0.01	mM
CP775146	DMSO	0.001	mM
DAPT	DMSO	0.01	mM
dmPGE2	Methylacetate	0.001	mM
EDA-A1	0.1% BSA in PBS	0.25	ug/ml
EGF	0.1% BSA in PBS	0.05	ug/ml
fedratinib	DMSO	0.01	mM
FGF10	0.1% BSA in PBS	0.01	ug/ml
FGF18	0.1% BSA in PBS	0.05	ug/ml
Forskolin	DMSO	0.01	mM
FR180204	DMSO	0.01	mM
Gefitinib	DMSO	0.01	mM
Gö6976	DMSO	0.001	mM
Gö6983	DMSO	0.005	mM
GSK0660	DMSO	0.001	mM
GW501516	DMSO	0.001	mM
GW6471	DMSO	0.001	mM
GW9662	DMSO	0.001	mM
H89	DMSO	0.005	mM
ICG-001	DMSO	0.005	mM

Ionomycin	DMSO	0.001	mM
IQ-1	DMSO	0.005	mM
JNK-IN-8	DMSO	0.001	mM
LDN193189	DMSO	0.00005	mM
LG100268	DMSO	0.001	mM
LY294002	DMSO	0.01	mM
M1 Fusion	DMSO	0.02	mM
Metformin	PBS	0.001	mM
ML264	DMSO	0.01	mM
OSM	0.1% BSA in PBS	0.1	ug/ml
PD0325901	DMSO	0.001	mM
Pluripotin	DMSO	0.001	mM
PMA	DMSO	0.00005	mM
Prolactin	0.1% BSA in PBS	0.5	ug/ml
Prostratin	DMSO	0.001	mM
ritlecitinib	DMSO	0.01	mM
Rspo1	0.1% BSA in PBS	0.25	ug/ml
Rspo2	0.1% BSA in PBS	0.2	ug/ml
Rspo3	0.1% BSA in PBS	0.2	ug/ml
ruxolitinib	DMSO	0.01	mM
SAG	DMSO	0.0001	mM
SB202190	DMSO	0.01	mM
SB203508	DMSO	0.01	mM
SB431542	DMSO	0.001	mM
SB590885	DMSO	0.001	mM
SHH	0.1% BSA in PBS	0.1	ug/ml
Somatostatin	PBS	0.00005	mM
SP100030	DMSO	0.005	mM
SP600125	DMSO	0.02	mM
SU5402	DMSO	0.01	mM
T5524	DMSO	0.01	mM
TGFb	0.1% BSA in PBS	0.005	ug/ml
TK216	DMSO	0.01	mM
tofacitinib	DMSO	0.01	mM
TUDC	EtOH	0.05	mM
VER115008	DMSO	0.005	mM
Verteporfin	DMSO	0.001	mM
WH-4-023	DMSO	0.001	mM
Wnt-C59	DMSO	0.00001	mM
Wnt3a	0.1% BSA in PBS	0.1	ug/ml
Wnt5a	0.1% BSA in PBS	0.1	ug/ml
Wnt7a	0.1% BSA in PBS	0.25	ug/ml
Wortmannin	DMSO	0.005	mM

XAV939	DMSO	0.001	mM
Yhhu3792	DMSO	0.001	mM
YK-4-279	DMSO	0.01	mM
