# Supplementary material for: Vitamin A resolves lineage plasticity to orchestrate stem cell lineage choices
Source: Science. Author manuscript; Available in PMC 2024 Jun 14. (PMC11177320; doi:10.1126/science.adi7342)
Supplement: Table S2 [file NIHMS1991969-supplement-Table_S2.pdf]

Antibody	Host	Supplier	Catalog #	Dilution IF	Western	Flow	CNR
ALDH1A2	rabbit	Sigma	HPA010022	1:200	---	---	---
ALDH1A3	rabbit	Thermo Fisher	PA5-29188	1:200	---	---	---
b-catenin	rabbit	Cell Signaling	8480	1:500	1:1,000	---	---
CD117-biotin	rat	eBiosciences	13-1171-82	---	---	1:250	---
CD140a-biotin	rat	eBiosciences	13-1401-82	---	---	1:200	---
CD200-PE	rat	eBiosciences	12-5200-80	---	---	1:200	---
CD31-biotin	rat	eBiosciences	13-0311-82	---	---	1:200	---
CD34-eF660	rat	eBiosciences	50-0341-82	---	---	1:100	---
CD45-biotin	rat	eBiosciences	13-0451-82	---	---	1:500	---
CUX1	rabbit	ProteinTech	11733-1-AP	1:200	1:1,000	---	---
GATA3	rat	eBiosciences	14-9966-82	1:200	1:1,000	---	---
GFP	chicken	Abcam	ab13970	1:2,000	---	---	---
HOXC13	rabbit	Sigma	HPA051634	1:250	1:1,000	---	---
a6-int-PE-Cy7	rat	eBiosciences	25-0495-82	---	---	1:500	---
b1-int-AF700	hamster	BioLegend	102218	---	---	1:500	---
b4-integrin	rat	BD	553745	1:400	---	---	---
KLF5	goat	R&D	AF3758	1:500	1:1,000	---	---
KRT10	rabbit	BioLegend	905401	1:200	1:1,000	---	---
KRT14	chicken	BioLegend	906001	1:1,000	1:1,000	---	---
KRT15	rabbit	Fuchs Lab	---	1:200	1:1,000	---	---
KRT6	guinea pig	Fuchs Lab	---	1:200	1:1,000	---	---
LEF1	rabbit	Cell Signaling	2230	1:200	1:1,000	---	---
pSMAD1	rabbit	Cell Signaling	13820	1:200	1:1,000	---	---
RARa	rabbit	Cell Signaling	62294S	1:200	1:1,000	---	---
RXRa	rabbit	Invitrogen	PA5-79945	---	---	---	1:50
RARg	rabbit	Cell Signaling	8965S	1:400	1:1,000	---	---
RARg	rabbit	Cell Signaling	3085S	---	---	---	1:50
RFP	rabbit	ProteinTech	5f8-100	1:1,000	---	---	---
Sca1-APC-Cy7	rat	BD	560654	---	---	1:1,000	---
SOX9	rabbit	Abcam	ab185966	1:250	---	---	---
SOX9	rabbit	Millipore	AB5535	1:200	1:500	---	---
Streptavidin-PerCP-Cy5.5	---	BD Biosciences	551419	---	---	1:1,000	---
TCF1	rabbit	Cell Signaling	2203	1:200	---	---	---
TCF3	guinea pig	Fuchs Lab	---	1:100	1:500	---	---
TCF4	rabbit	Cell Signaling	2569	1:200	1:1,000	---	---
Tubulin	mouse	Sigma	T5168	---	1:5,000	---	---
