# Supplementary material for: Vitamin A resolves lineage plasticity to orchestrate stem cell lineage choices
Source: Science. Author manuscript; Available in PMC 2024 Jun 14. (PMC11177320; doi:10.1126/science.adi7342)
Supplement: Table S3 [file NIHMS1991969-supplement-Table_S3.pdf]

Gene	Forward Sequence	Reverse Sequence
Axin2	actgaccgacgattccatgt	ctgcgatgcatctctctctg
Cux1	tgctctggagaaagagcaga	tgctcgcttcctccagttt
Cyp26b1	gaatgtgcgcaagatcctactg	gatctgccgagcttggatg
Ets2	gattctgtctcccatgactcg	gtcgttgctccttttggaag
Gata3	aggcaaccacgtcccgtcct	agatccgtgcagcagaggcg
Hoxc13	gggctatggttacccatttgg	ctggaggacaggtcgtcac
Id1	agtctgaagtcgggaccac	gatcgtcggctggaacac
Klf5	ggctctccccgagttcacta	attactgccgctggtttgtc
Lef1	ccgagatcagtcatcccgaag	ggcttgtctgaccacctcat
Lgr5	gcaaacttcccagagctcaa	gttgccgtcgtctttattcc
Nfatc1	aacgccctgaccaccgatagcact	cccggctgccttccgtctcata
Nfib	tgaggcagcttcacctacag	aggatgggtctcttgcgctta
Ppib2	gtgagcgcttcccagatgaga	tgccggagtcgacaatgatg
Sox9	agaaagaccaccccgattacaagt	cggcggaccctgagattg
Tbx1	ctcgtgagtgcctttgctc	cggagtcacggtcgaact
Tcf7l1	tggtcaacgaatcggagaat	tcacttcggcgaaatagtcg
Tcf7l2	ctccacagctcaaagcatca	caccaccttcgctctcatct
